# A lipid metabolism–based prognostic risk model for HBV–related hepatocellular carcinoma

**DOI:** 10.1186/s12944-023-01780-9

**Published:** 2023-04-01

**Authors:** Lili Zhou, Shaohuai Xia, Yaoyao Liu, Qiang Ji, Lifeng Li, Xuan Gao, Xiaodi Guo, Xin Yi, Feng Chen

**Affiliations:** 1 Cancer Center, Beijing Tiantan Hospital, Capital Medical University, No. 119 Nansihuan West Road, Fengtai District, Beijing, 100070 China; 2 Beijing Fuzheng Cancer Hospital, No. 20 Jinghai 3rd road, Yizhuang Economic and Technological Development Zone, Beijing, 100070 China; 3 Beijing GenePlus Genomics Institute, Beijing, 102205 China; 4 State Key Laboratory of Microbial Resources, Institute of Microbiology, Chinese Academy of Sciences, Beijing, 100101 China; 5 Shenzhen GenePlus Clinical Laboratory, ShenZhen, 518122 China

**Keywords:** HBV, HCC, Lipid metabolism, Prognostic risk model, Genomic instability, TME

## Abstract

**Background:**

Up to 85% of hepatocellular carcinoma (HCC) cases in China can be attributed to infection of hepatitis B virus (HBV). Lipid metabolism performs important function in hepatocarcinogenesis of HBV–related liver carcinoma. However, limited studies have explored the prognostic role of lipid metabolism in HBV–related HCC. This study established a prognostic model to stratify HBV–related HCC based on lipid metabolisms.

**Methods:**

Based on The Cancer Genome Atlas HBV–related HCC samples, this study selected prognosis-related lipid metabolism genes and established a prognosis risk model by performing uni- and multi-variate Cox regression methods. The final markers used to establish the model were selected through the least absolute shrinkage and selection operator method. Analysis of functional enrichment, immune landscape, and genomic alteration was utilized to investigate the inner molecular mechanism involved in prognosis.

**Results:**

The risk model independently stratified HBV-infected patients with liver cancer into two risk groups. The low–risk groups harbored longer survival times (with *P* < 0.05, log–rank test). *TP53*, *LRP1B*, *TTN,* and *DNAH8* mutations and high genomic instability occurred in high–risk groups. Low–risk groups harbored higher CD8 T cell infiltration and *BTLA* expression. Lipid–metabolism (including “Fatty acid metabolism”) and immune pathways were significantly enriched (*P* < 0.05) in the low–risk groups.

**Conclusions:**

This study established a robust model to stratify HBV–related HCC effectively. Analysis results decode in part the heterogeneity of HBV–related liver cancer and highlight perturbation of lipid metabolism in HBV–related HCC. This study’s findings could facilitate patients’ clinical classification and give hints for treatment selection.

**Supplementary Information:**

The online version contains supplementary material available at 10.1186/s12944-023-01780-9.

## Background

Accounting for 85–90% of primary liver cancer worldwide [1], hepatocellular carcinoma (HCC) ranks as the 4th attribution of carcinoma–related death [2]. In China, up to 85% of live carcinoma with infection of hepatitis B virus (HBV) [3]. Poor recovery and high recurrence characterize HBV–related HCC cases [4]. HBV–induced HCC has high intratumoral heterogeneity, and current therapy has obtained rare achievements [5]. Exploring this heterogeneity to effectively stratify patients with potentially different survival rates and underlying molecular mechanisms could inform prognosis and treatment selection.

Lipid metabolism is an energy source that can support rapid cell division, and it functions in micro–environmental adaptation and cell signaling in HCC [6, 7]. Perturbation of lipid metabolism underlies tumorigenesis [8, 9]. In HBV–related HCC, lipid metabolism is a potential driving force and its perturbation plays an important role in hepatocarcinogenesis [6, 7]. In turn, HBV infection can affect pathways of lipid metabolism, including “fatty acid metabolism pathway” and “phospholipid and cholesterol metabolism pathway”, which ultimately result in metabolic dysregulation [10]. Thus, it is crucial to investigate the function of lipid metabolism and its potential influence on patients’ survival in HBV+ HCC.

Several studies have developed prognostic biomarkers based on lipid metabolism for HCC [11, 12], but have ignored the high heterogeneity of HBV–related HCC. Little attention has been paid to HBV–related HCC, and no specific prognostic model based on lipid metabolism has been developed.

Another study discovered a significant correlation between immune cells of the tumor microenvironment (TME) and lipid-metabolism, potentially indicative of lipid metabolism’s immune regulation function [8]. Chronic HBV infection can induce immune imbalance [13] and immune suppression [14]. HBV–positive HCC tumors have significantly lower expression levels of NK and CD8+ T cells than HBV-negative HCC [15]. These previous findings suggest that viral infection might shape a unique TME and that lipid metabolism might modulate the immune system in HBV–related liver cancer.

For the first time, this study explored the critical prognostic role of lipid metabolism in HBV-related HCC. The risk model is a new independent prognostic tool to stratify HBV–related HCC patients. The molecular characteristics that differ between the two risk groups might be potential targets for alternative immune therapies.

## Material and method

### Data collection and preprocessing

RNA–seq mRNA expression profiles with matching clinic data of HBV-infected patients were acquired from three cohorts: The Cancer Genome Atlas (TCGA) database, Gao et al. study [16] and Roessler et al. study [17]. The HBV–related samples of the TCGA database were obtained as the training set, and the HBV–related samples were obtained according to two previous reports: 44 and 87 TCGA HBV–related samples, respectively [18, 19]. The final sample union of 44 and 87 is 112. Thus, the training set includes 112 unique samples. The risk model was validated in two independent datasets: 159 HBV–related samples with overall survival (OS) information collected from the Gao et al. study; 55 active HBV replication chronic patients with OS and relapse-free survival (RFS) information derived from a clinical trial cohort of Roessler et al. study, in which patients underwent radical resection with liver cirrhosis-related to HBV or HBV infection history [17].

Patients with less than 10 days of OS were eliminated because HCC might not have been the cause of death. In addition, 558 genes included in 21 lipid–metabolism pathways derived from the Kyoto Encyclopedia of Genes and Genomes (KEGG) were included in this study by reference to Hao et al. [8]. To eliminate batch effects, gene–expression levels were z-score–normalized and subsequent analyses were based on the normalized data.

### Prognostic risk model construction and validation

Uni-variate and multi-variate Cox regression methods were conducted to screen for lipid–metabolism genes related to prognosis to include in the risk model, The regression analysis filtered out 45 prognosis–related lipid–metabolism genes. Then Least Absolute Shrinkage and Selection Operator (LASSO) were used to shrink variables to optimize the model (Fig. S1). Eleven genes were selected from the 45 prognosis–related genes through the LASSO algorithm with “lambda.min” as the shrinkage variable. The risk model was established using the 11 genes with multi-variate Cox regression coefficients as the risk parameters. For each sample S_i_, a new score was calculated as follows:

$${S}_i\ score= LPCAT{3}_{{{\exp}}_i}\ast \left(-1.227194e-05\right)+ CAMKK{2}_{{{\exp}}_i}\ast \left(1.575379e-05\right)+ GGT{5}_{{{\exp}}_i}\ast \left(4.809144e-06\right)+ PLD{4}_{{{\exp}}_i}\ast \left(-6.965001\textrm{e}-05\right)+ ADCY{5}_{{{\exp}}_i}\ast \left(-3.380608e-05\right)+ PLD{1}_{{{\exp}}_i}\ast \left(2.600955e-05\right)+ UGT{8}_{{{\exp}}_i}\ast \left(3.719908e-04\right)+{LIPF}_{{{\exp}}_i}\ast \left(2.558418e-03\right)+{TNF}_{{{\exp}}_i}\ast \left(-2.889406e-04\right)+ MMP{1}_{{{\exp}}_i}\ast \left(-3.260970e-05\right)+G6{PC}_{{{\exp}}_i}\ast \left(-2.651959e-07\right),$$

in which exp_i_ indicates gene expression values in the sample S_i_. The new score of each sample, S_i_ score, was defined as the prognostic risk value. Subsequently, the “surv_cutpoint” function (the method in the R package: “survminer”) was utilized to determine the optimal threshold of patients’ prognostic risk scores to separate samples into two different risk groups (high-risk group: patients with scores more than the optimal threshold value; low-risk group: patients with scores less than the optimal threshold value). Additionally, the importance of 11 genes was calculated in the risk model in each cohort using the varImp function to evaluate their prognostic contributions (Fig. S2). Survival was compared between two different groups by Kaplan–Meier method. *P* value was examined by Log–rank test.

### Pathways enrichment analysis

Through “gseKEGG”, pathway enrichment of KEGG and Hallmark were analyzed. The “clusterProfiler” R package was utilized for Gene Set Enrichment Analysis. The significant cut–off criteria of *P* is less than 0.05, the absolute value of the normalized enrichment score is not more than 1, and the cutoff of false discovery rate is not more than 0.25.

### Intratumoral immune cell infiltration

Intratumoral immune–cell infiltration levels were quantified using the xCell, cibersort, and ESTIMATE methods in the “IOBR” and “estimate” R packages. Intratumoral immune infiltration was evaluated with the “gsva” function based on the expression of corresponding metagenes derived from an immunogenomic study [20]. The clustering was based on the k–means algorithm. The intergroup comparisons were carried out using Fisher’s exact test. The visual graphs of the analysis results were boxplots and heatmaps.

### Genetic alteration analysis

Mutation analysis of the TCGA HBV–related HCC cohort was conducted to compare genetic alterations in two risk groups. Fisher’s exact test was used to assess the statistical significance. The genetic alteration analysis results were visualized through the maftools R package [21]. The MATH value in each tumor was calculated as described in a previous study [22]: MATH = 148.26 × MAD/median. MAD is the median absolute deviation, and median denotes the median mutation allele fragments value at cancer–specific mutation locus. Tumor mutation burden (TMB) values were derived from the R package: “TCGAmutations”.

### Statistical analyses

Statistical analyses were conducted in R software with version 4.1.0. The clinical features comparison between the two groups were examined by Fisher’s exact test. Mutation differences were compared by utilizing Fisher’s exact test. Survival analysis was conducted by Kaplan–Meier method and *P* values were examined by Log–rank test. Other intergroup differences were compared using the Wilcoxon rank–sum test. The significant statistics criterion of *P* is not more than 0.05.

## Results

### Lipid metabolism–related prognostic signature identification

Uni- and multi-variate Cox regression methods were implemented on 558 lipid metabolism–related genes to identify prognosis–related ones in the TCGA HBV–related HCC dataset with disease–free interval (DFI) survival data. HBV–related HCC showed heterogeneity in OS. Within 2 years, HBV+ HCC patients had poorer survival than HBV– patients in the TCGA HBV–related live cancer cohort, whereas after 2 years, the survival trend was the opposite (Fig. S3). Considering lipid metabolism’s important function in tumorigenesis, this study developed a lipid metabolism-based prognostic model of HBV+ HCC. In total, 59 genes were related to prognosis in uni-variate Cox regression analysis. Next, these genes were analyzed using multi-variate Cox regression and 43 genes significantly (*P*  <   0.05) related to survival were selected. The coefficients of multi-variate Cox regression were set as the risk parameters. Finally, these 43 genes were screened using LASSO analysis (Fig. S1) to identify the most prognostic genes, and 11 genes were identified to include in the prognostic risk model.

The mRNA expression patterns and corresponding protein expression in HBV+ liver carcinoma and normal tissue adjacent to tumor (NAT) of these 11 genes were examined. In the TCGA HBV–related HCC cohort, eight genes showed significantly different expression levels between NAT and HBV+ HCC groups. Seven of the genes (*MMP1* being the exception) showed significantly lower expression in HBV+ HCC (*P* < 0.05; Fig. S4A). In the Gao et al. cohort, nine genes showed significantly different expression levels between HBV+ HCC and NAT. Seven genes had lower expression in HBV+ live cancer compared with NAT samples, with *LPCAT3* and *MMP1* being the exceptions (*P* < 0.05; Fig. S4B). Protein expression data were unavailable for the 11 markers in the TCGA HBV–related HCC cohort but available for 5 of the 11 markers in the Gao et al. cohort. All five markers’ protein expression levels were significantly lower in HBV+ live cancer than in NAT samples (*P* < 0.001; Fig. S4C).

### Prognostic risk model construction and validation of predictive performance

After identifying lipid metabolism–related prognosis signatures, the prognostic risk model based on these 11 markers was constructed. For each sample, the risk value was calculated based on the product of risk-score parameters and the expression value of the 11 genes (see Materials and methods). On the basis of risk value, samples were divided into two risk groups (named high– and low–risk groups). The clinical features of the two risk groups in both cohorts were listed in Tables 1 and 2. In the TCGA HBV–related HCC patients, tumor stage distribution was significantly different between the two groups. The high–risk patients harbored larger fractions of stage II and III tumors (Table 1, 31.8 and 54.5%, respectively, *P* = 0.0001216) and a larger proportion of stage I in low–risk tumors (Table 1, 60%, *P* = 0.0001216). In Gao et al. cohort, the distribution of Barcelona clinic liver cancer stage (BCLC) and the distribution of tumor size was significantly different in the two risk groups, with larger proportions of small tumors (Table 2, *P* = 0.002239, <= 5.5 cm; 75%) and stage A tumors of BCLC (Table 2, *P* = 0.009862, 61.1%) in the low–risk patients.

**Table 1:** Clinical pathological characteristics of patients and the correlation between those parameters and overall survival in two risk groups for the TCGA cohort. BCLC, Barcelona clinic liver cancer stage

Variable	High–risk	Low–risk	*P*
	*n* = 22 (21.6%)	*n* = 80 (80.4%)	
Sex			0.3907
Male	19 (86.4%)	61 (76.3%)	
Female	3 (13.6%)	19 (23.8%)	
Age, years			0.1106
> 60	3 (13.6%)	26 (32.5%)	
< =60	19 (86.4%)	54 (67.5%)	
Tumor stage			0.0001216
I	3 (13.6%)	48 (60%)	
II	7 (31.8%)	17 (21.3%)	
III	12 (54.5%)	14 (17.5%)	
IV	0 (0%)	1 (1.3%)	
Histological grade			0.5494
G1	3 (13.6%)	5 (6.3%)	
G2	7 (31.8%)	35 (43.8%)	
G3	11 (50%)	34 (42.5%)	
G4	1 (4.5%)	6 (7.5%)

**Table 2:** Clinical pathological characteristics of patients and the correlation between those parameters and overall survival in two risk groups for the Gao et al. cohort. BCLC, Barcelona clinic liver cancer stage

Variable	High–risk	Low–risk	*P*
	*n* = 123 (77.4%)	*n* = 36 (22.6%)	
Sex			0.3465
Male	101 (82.1%)	27 (75%)	
Female	22 (17.9%)	9 (25%)	
Age, years			0.101
> 60	33 (26.8%)	15 (41.7%)	
< =60	90 (73.2%)	21 (58.3%)	
Tumor number			0.8322
> 1	32 (26%)	10 (27.8%)	
< =1	91 (74%)	26 (72.2%)	
Tumor size, cm			0.002239
> 5.5	67 (54.5%)	9 (25%)	
< =5.5	56 (45.5%)	27 (75%)	
Lymph node metastasis			0.4027
yes	1 (0.8%)	1 (2.8%)	
no	122 (99.2%)	35 (97.2%)	
BCLC stage			0.009862
A	46 (37.4%)	22 (61.1%)	
B	47 (38.2%)	5 (13.9%)	
C	30 (24.4%)	9 (25%)	
TNM stage			0.3525
I	71 (57.7%)	20 (55.6%)	
II	9 (7.3%)	5 (13.9%)	
III	42 (34.1%)	10 (27.8%)	
IV	1 (0.8%)	1 (2.8%)	

**Fig. 1:**
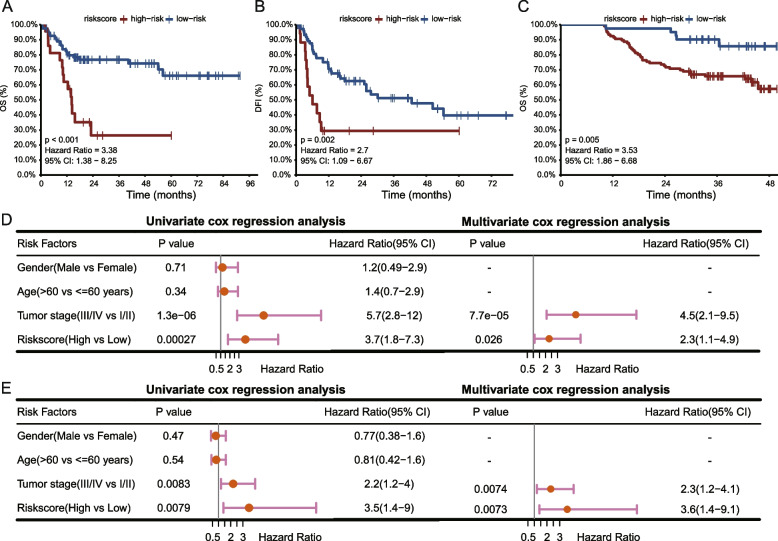
Predictive performance and independent prognostic capacity of the risk model. **A**, **B** Kaplan–Meier survival curves of OS and DFI stratified by the risk model of the TCGA HBV–related HCC tumors. **C** OS Kaplan–Meier survival curves for HBV–related HCC tumors stratified by the risk model in the Gao et al. cohort. **D**, **E**) Uni- and multi-variate Cox regression methods results based on OS of the TCGA and Gao et al. cohorts, respectively

Subsequently, survival analysis was utilized to determine whether this risk model could effectively stratify patient survival in the TCGA HBV–related HCC and another two independent cohorts, including Gao et al. cohort and Roessler et al. cohort. Kaplan–Meier curve showed a significantly worse prognosis of high–risk patients in the TCGA HBV–related HCC dataset (Fig. 1 A, B; OS: *P* < 0.001, DFI: *P* = 0.002). The Gao et al. cohort consistently showed the same trend (Fig. 1 C; OS: *P* = 0.005), in which low–risk patients exhibited better survival. This study obtained 55 active HBV replication chronic patients with overall survival (OS) and RFS information from the Roessler et al. cohort. The risk model divided the cohort into two risk groups, with survival analysis showing a significant difference in RFS between the two groups. In addition, the high-risk groups had poorer survival (Fig. S5A; *P* = 0.033, HR = 2.12) than low-risk group. The OS showed survival probability difference among different follow-up periods (Fig. S5B; *P* = 0.47, HR = 1.35), in which high-risk groups showed poorer OS after about 32 months but better OS within about 32 months.

### The risk model can independently predict prognostic for HBV–related HCC

Survival analytic results showed the prognostic capacity of the risk model. Next, to explore whether the model can independently predict survival, uni- and multi-variate Cox regression analysis were carried out in the TCGA and Gao et al. datasets. The uni-variate Cox regression method showed the tumor stage and the risk score significantly associated with OS (Fig. 1 D; risk score: *P* = 0.00027, hazard ratio [HR] = 3.7; tumor stage: *P* = 1.3E–06, HR = 5.7) in the TCGA HBV–related HCC cohort. Risk score (Fig. 1 E; *P* = 0.0079, HR = 3.5, 95% CI = 1.4–9) and tumor stage (Fig. 1 E; *P* = 0.0083, HR = 2.2) were significantly related to OS in the Gao et al. also. Multi-variate Cox regression showed risk score and prognosis remained significantly related after adjustment of other clinic factors in both the TCGA (Fig. 1 D; *P* = 0.026, HR = 2.3) and Gao et al. (Fig. 1 E; *P* = 0.0073, HR = 3.6) cohorts. Uni- and multi-variate Cox regression were also conducted for DFI in the TGCA dataset and relapse–free survival (RFS) in the Gao et al. cohort, respectively (Fig. S6). The results show that risk score can predict prognosis for HBV–related HCC, independent of other clinicopathological factors, and that lower risk scores indicate longer survival times.

### Decoding TME context in two risk groups

**Fig. 2:**
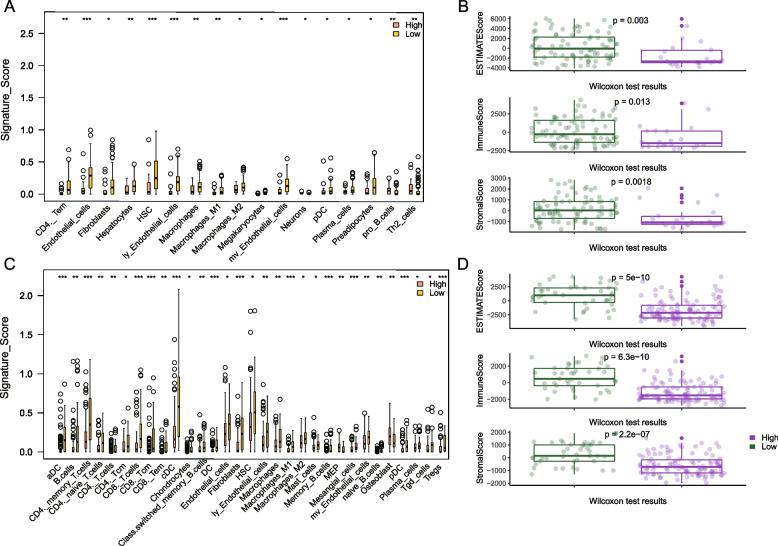
Immune–cell infiltration. Immune–cell infiltration differences between two groups of the TCGA HBV–related HCC dataset (**A**, **B**) and Gao et al. cohort (**C**, **D**) quantified by xCell and ESTIMATE. * a single asterisk means *P* < 0.05, ** two asterisks mean *P* < 0.01, and *** three asterisks mean *P* < 0.001

To decode the immune-cell landscape of two different groups, the infiltration of immune–cells was quantified (see Materials and methods). Results using the xCell methodology indicated that in the low–risk patients in each dataset, endothelial cells, macrophages, M1 and M2 macrophages all accounted for larger proportions (Fig. 2 A, TCGA HBV–related HCC cohort: *P* = 3.43E–05, *P* = 0.0061, *P* = 0.0030, and *P* = 0.03; Fig. 2 C, HBV–related HCC in the Gao et al. dataset: *P* = 0.016, *P* = 0.0059, *P* = 9.57E–05, and *P* = 0.031). In the low–risk patients, Plasmacytoid dendritic cells (pDCs) harbored higher infiltration (TCGA HBV–related HCC cohort: *P* = 1.50E–02; Gao et al. cohort: *P* = 6.31E–05).

According to the cibersort method, the low-risk patients harbored significantly larger proportions of CD8 T cells and dendritic-resting cells (TCGA HBV–related HCC dataset: *P* = 0.039, *P* = 0.024, respectively; Gao et al. dataset: *P* = 0.00018, *P* = 0.0057). In the low–risk patients of the Gao et al. dataset, activated mast cells and M1 macrophages harbored significantly higher expression levels (*P* = 0.0053, *P* = 0.047, respectively). M0 macrophages exhibited a converse trend, with higher proportions in the high–risk patients (TCGA HBV–related HCC cohort: *P* = 0.29, Gao et al. cohort: *P* = 0.00058; Fig. S7).

Consistently, StromalScore, ImmuneScore, and ESTIMATEScore quantified by the ESTIMATE method showed a higher score in low–risk cases (Fig. 2 B, D; TCGA HBV–related HCC cohort: *P* = 0.0018, *P* = 0.013, *P* = 0.003, respectively; *P* = 2.2E–07, *P* = 6.3E–10, *P* = 5E–10, respectively in Gao et al. cohort; Wilcoxon rank–sum test). Overall, immune cell infiltration was significantly greater in the low–risk groups.

**Fig. 3:**
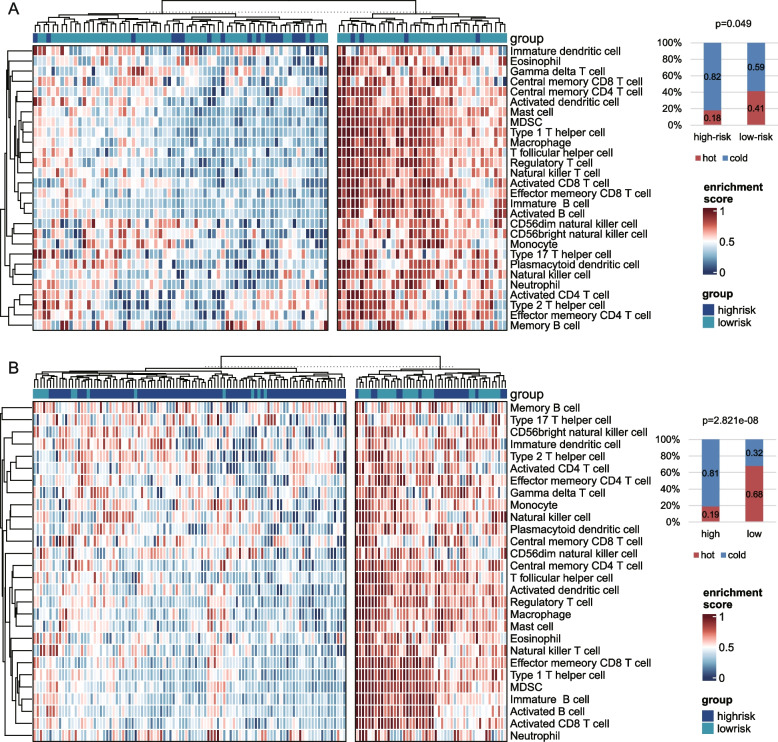
Heatmap of 28 immune–cell infiltration. Infiltration Heatmap of immune cells in the TCGA HBV–related HCC dataset (**A**) and Gao et al. (**B**) cohorts. The row means the type of immune cell. The column corresponds to each sample. The upper right bar chart in each panel shows the proportions of “hot” and “cold” immune states in two risk groups

The intratumoral immune composition was explored more comprehensively by assessing 28 immune–cell subpopulations reported in a pan–cancer immunogenomic analysis [20]. In general, the high–risk groups showed “cold” tumors with lower immune cells expression levels, whereas the low–risk patients had “hot” tumors (Fig. 3; TCGA HBV–related HCC cohort: *P* = 0.049, Gao et al. cohort: *P* = 2.821E–08; Fisher’s exact test).

### Function enrichment characterizes high–risk and low–risk patients

**Fig. 4:**
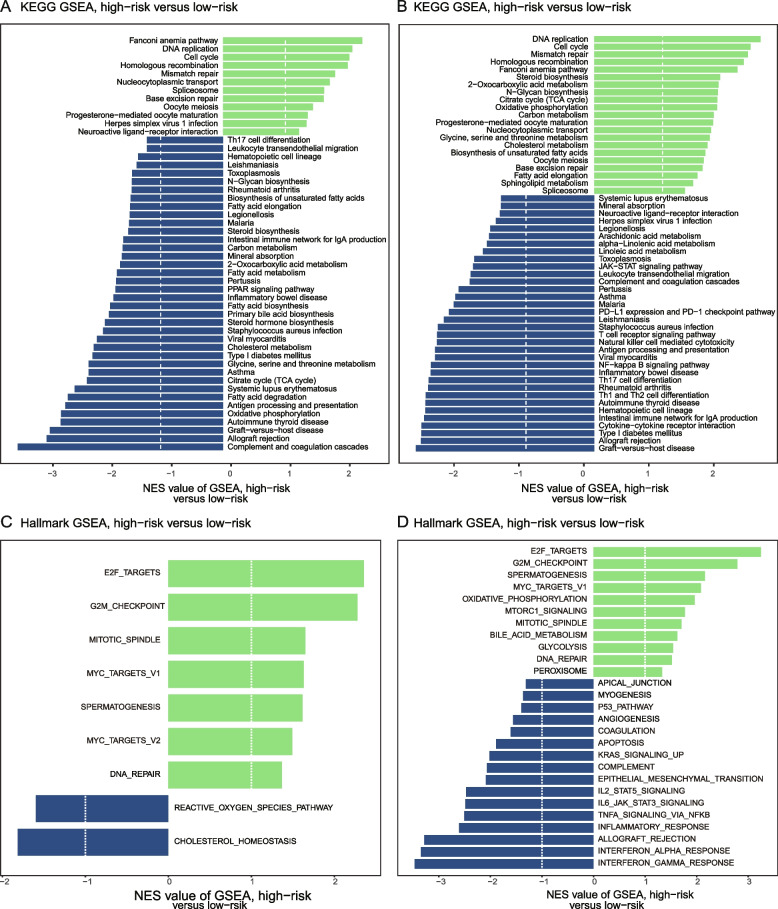
Functional enrichment analysis. **A**, **B** Representative and significantly enriched KEGG pathways in two risk groups of the TCGA and Gao et al. cohorts. **C**, **D** Hallmark pathway analysis results in two risk groups of the TCGA and Gao et al. cohorts. Green and blue indicate enriched pathways in high– and low-risk patients with significant *P*

This study performed function analysis to explore the intrinsic biological mechanisms between two risk groups and the interaction of lipid-metabolism with the immune microenvironment. The overlapping pathways were exhibited, including lipid metabolism–related and immuno–related pathways among all the significant enrichment pathways in the two datasets (Fig. 4). The detailed and complete results are available in Fig. S8. Collectively, the results revealed that in the high–risk groups, “Homologous recombination”, “Cell cycle”, “DNA replication”, “Mismatch repair” and “Base excision repair” pathways significantly enriched (Fig. 4). “Antigen processing and presentation”, “Leukocyte transendothelial migration”, and “Th17 cell differentiation” pathways were shared among low–risk patients in each dataset (Fig. 4). In high–risk patients of both cohorts, results of Hallmark analysis also showed the “DNA repair” pathway significantly enriched. Metabolic pathways consisting of “Fatty acid biosynthesis”, “Fatty acid degradation”, “Fatty acid metabolism”, “PPAR signaling pathway”, and “Biosynthesis of unsaturated fatty acids” significantly enriched in the low–risk group of the TCGA HBV–related HCC cohort (Fig. 4). Additionally, pathways of immune, including “Leukocyte transendothelial migration”, “Antigen processing and presentation”, and “Th17-cell differentiation” showed significant enrichment in low–risk cases of the TCGA HBV–related HCC dataset (Fig. 4). In the Gao et al. dataset, low–risk enriched more immune pathways, involving “Leukocyte transendothelial migration”, “PDL1 expression and PD1 checkpoint pathway in cancer”, “Th1 and Th2 cell differentiation”, “B cell receptor”, “Natural killer cell mediated cytotoxicity”, “Th17 cell differentiation”, and “T cell receptor”, and “Antigen processing and presentation” (Fig. 4).

**Fig. 5:**
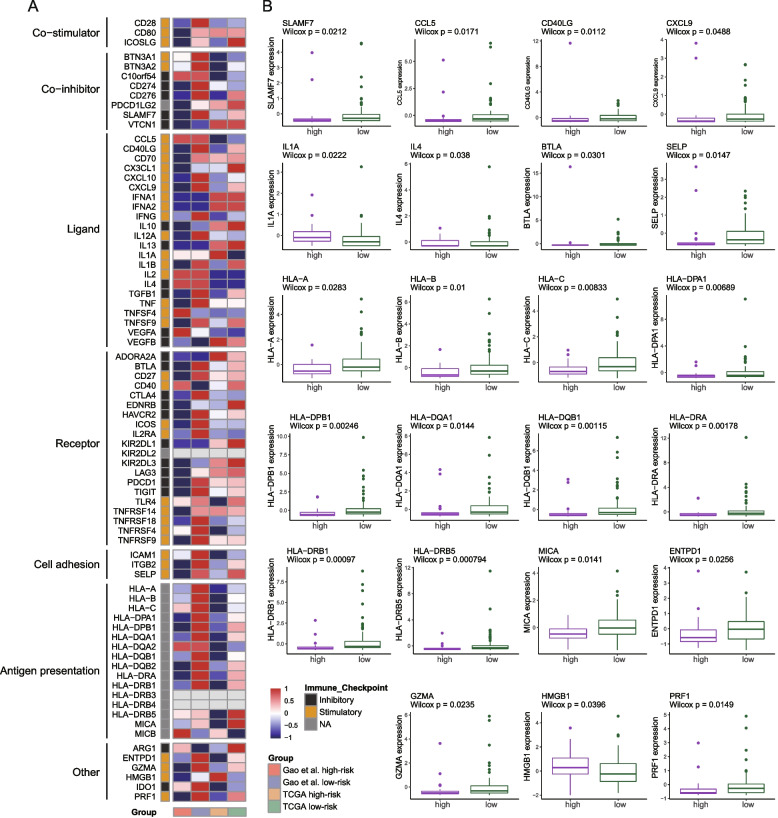
Immune gene expression differences between two risk groups. **A** Average expression heatmap of 78 immune genes. The row and column represent a kind of gene and a group. **B** Box plots of genes with significantly different expression levels between two risk groups of the TCGA HBV–related HCC cohort

The immune genes’ expression [23] was also examined in two groups. A heatmap (Fig. 5 A) outlines the expression landscape of those genes in the two cohorts, and boxplots show the significantly expressed results between two risk groups (Fig. 5 B, *P* < 0.05, TCGA HBV–induced HCC cohort; Fig. S9, *P* < 0.05, Gao et al. cohort; Wilcoxon rank sum test). In line with the immune–related pathways enrichment results observed in low–risk groups, 17 immune–related genes, including a co–inhibitor (*SLAMF7*), ligands (*CD40LG*, *CXCL9*), a receptor (*BTLA*), a cell adhesion (*SELP*), molecules involved in antigen presentation, *ENTPD1*, *GZMA*, and *PRF1* expressed significantly higher in low-risk patients of both cohorts (Fig. 5 B, Fig. S9). In low–risk groups, the immune checkpoint gene *BTLA* had significantly elevated expression in both cohorts. In the Gao et al. cohort, *PDCD1* and *CD274* had significantly increased expression (Fig. S9).

### More genetic mutations occurred in high–risk groups

**Fig. 6:**
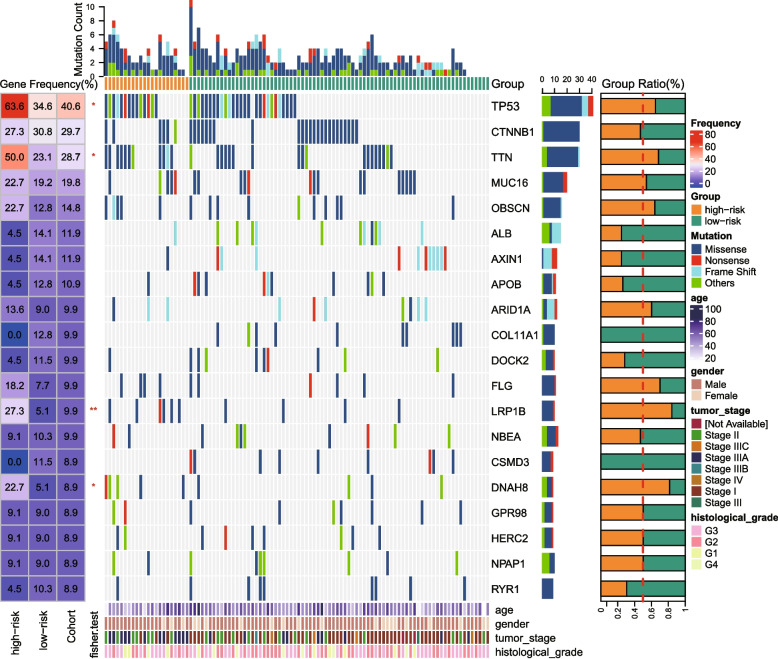
The gene mutation landscape of the TCGA HBV–related HCC cohort

Further exploration of gene mutation levels was conducted in HBV+ HCC. As indicated in Fig. 6, the 20 most frequently altered genes were identified. Mutation of *TP53* and *LRP1B* was identified in 40.6 and 9.9%, respectively, in the cohort of TCGA. In the high–risk patients, *TP53* and *LRP1B* mutations rate had significantly higher levels (63.6 and 27.3%, respectively) (Fig. 6; *P* < 0.05). *TTN* and *DNAH8* showed mutation frequencies of 28.7 and 8.9%, respectively, in the whole cohort. Similar to *TP53* and *LRP1B*, the mutation rate of *TTN* and *DNAH8* were significantly higher in high–risk patients. To further explore mutation numbers and genetic heterogeneity between the two groups, we examined TMB and MATH values and high–risk cases showed higher median TMB and MATH values (Fig. S10A–B; median TMB: 2.29 vs. 2.16, median MATH value: 86.5 vs. 83), but the differences were not significant.

Mutation landscape of two risk groups. The top bar chart shows the gene mutation counts of each sample. The table on the left indicates gene mutation frequency in two risk groups and the whole cohort. The heatmap portrays the gene mutation landscape, in which different mutation types were annotated with different colors. The bar plot next to the heatmap indicates the mutation type proportions in all samples for each gene. The bar graph on the far right exhibits mutation proportion in two risk groups for each gene. The bottom bar chart shows clinical characteristics. * a single asterisk means *P* < 0.05, ** two asterisks mean *P* < 0.01.

## Discussion

The assessment of intratumoral immune–cell infiltration revealed that high–risk patients tended to be cold tumor whereas low–risk groups had hot tumors. Significantly higher expression of *BTLA* was found in low–risk groups. Cai et al. revealed that upregulation of *BTLA* can restrict T–cell responses [24], suggesting that blockage of *BTLA* might be a potential therapeutic option for low–risk groups. In addition, in the Gao et al. cohort, immune checkpoint genes’ expression including *PDCD*, *CD274* and *CTLA4*, was significantly elevated in low–risk groups. A previous study found that immune checkpoint genes, including those three genes, showed significantly increased expression in “immune high” HBV patients [25], which is consistent with our results.

Function analysis showed DNA replication and repair–related pathways significantly enriched in high-risk patients, which might indicate that more genomic alterations occurred in these patients. Consistently, the genomic mutation analysis revealed more gene mutations (including in *TP53*, *LRP1B*, *DNAH8*, and *TTN)* in high–risk cases, suggestive of higher genomic instability. Consistent with a previous report that mutations of *TP53* and *LRP1B* indicate poor prognosis [26], the high–risk groups had more mutations in these genes and showed shorter survival times. As reported by Dong et al., mutated *DNAH8* is associated with a worse prognosis [27] and shorter disease–free survival. Similarly, this study’s high–risk groups had a higher frequency of *DNAH8* mutation harboring and reduced OS and DFI. In addition, a published study identified *TP53* and *TTN* as among the top ten genes with high mutation rates and cancer drivers of HBV–related HCC [19]. All results indicated high–risk groups harbored high genomic instability and might be sensitive to DNA–damaging therapy.

### Comparing with other studies and contribution to existing knowledge

Given the high heterogeneity of HBV–induced HCC and the limited achievements of current therapies, novel strategies to stratify patients could be valuable to inform prognosis and potentially guide clinical therapy. Dysregulation of lipid metabolism can lead to tumorigenesis, and there is complicated crosstalk between immune response and lipid-metabolism [8, 9]. In HBV–related liver carcinoma, lipid metabolism functions as the driving force and affects hepatocarcinogenesis [6, 7]. HBV infection, in turn, can affect lipid metabolism. However, most studies have focused on exploring prognostic markers for all HCC patients without considering HBV-related HCC’s heterogeneity. This study explored the lipid metabolism’s prognostic function in HBV–related liver cancer patients and the molecular mechanisms between different risk groups.

### Study strengths and limitations

For the first time, based on lipid metabolism–related genes, a prognostic model was established for HBV–related HCC. The lipid metabolism markers showed downregulated in HBV–related liver cancer. The risk model can effectively stratify patients into two risk groups and was validated through two independent datasets. The low–risk groups showed significantly better survival. And the model can also serve as an independent prognostic tool. Furthermore, TME, genomic alteration, and the intrinsic association between the immune microenvironment and lipid-metabolism were explored between the two groups. High–risk groups showed lower immune-cell infiltration levels. In the low–risk group, high macrophages and CD8 T cells infiltration levels and elevated BTLA expression suggested that alternative immune therapy might hold promise for these patients. The results showed higher genomic instability in high–risk groups, indicating that these patients might be more sensitive to DNA–damaging treatment.

Certain limitations of this study should be noted. Although the risk model showed robust performance, the validation was conducted in two independent datasets because limited HBV–related HCC datasets, including transcriptome and survival information, are available. Thus, the risk model warrants further investigation and validation in more independent cohorts. In addition, corresponding potentially sensitive drugs of high- and low-risk groups suggested according to distinct TME, genomic and transcriptomic characteristics need to be further explored and validated in both molecular mechanism and clinical trial levels. Although this study revealed simultaneous enrichment of immune and metabolism pathways in the low–risk groups, the inner mechanism by which metabolism affects the immune microenvironment, or vice versa, needs further exploration.

## Conclusions

This work highlights the perturbation of lipid metabolism in HBV–related HCC and partly shows the inner molecular and prognosis heterogeneity of HBV–related liver carcinoma patients. The risk model can facilitate stratifying clinical HBV–related liver carcinoma patients. The risk model based on lipid metabolism can independently predict prognosis with robust performance and the low-risk group showed significantly better survival than the high-risk group. Multi-omics analysis showed distinct characteristics of two risk groups and the key characteristics exhibited in two risk groups might lead toward the development of different therapeutic methods. The low-risk group patients showed higher CD8 T cell infiltration levels and elevated expression of the immune checkpoint gene BTLA, which implies that alternative immune therapy might hold promise for the low-risk group patients. The high–risk groups exhibited higher genomic instability with more gene mutation and enriched DNA replication and repair–related pathways, suggesting that the high-risk group might be more sensitive to DNA–damaging treatment.

## Supplementary Information

**Additional file 1: Fig. S1.** Feature selection by LASSO logistic regression. **Fig. S2.** The prognostic contributions of eleven marker genes in the risk model. **Fig. S3.**Survival difference analysis between hbv + and hbv- HCC patients in the TCGA cohort. **Fig. S4.** Expression patterns comparison of 11 marker genes between hbv + HCC and normal samples. (A-B) The mRNAs expression level of 11 genes in the TCGA and Gao et al. cohorts, respectively. (C) The protein expression level of 11 genes in Gao et al. cohort. **Fig. S5.** Survival analysis of high- and low-risk groups. **Fig. S6.** Independent prognostic prediction analysis of our risk model. **Fig. S7.** Immune cells infiltration difference between high- and low-risk groups quantified by cibersort algorithm. **Fig. S8.** Functional enrichment analysis. **Fig. S9.** Analysis of immune gene expression difference in high- and low-risk groups. **Fig. S10.** TMB and intratumor genetic heterogeneity difference between high- and low-risk groups.

## Data Availability

The data analyzed are publicly available in published articles which were cited in this study and the TCGA database (https://portal.gdc.cancer.gov/).

## Abbreviations

OS: Overall survival
TCGA: The Cancer Genome Atlas
LASSO: Least Absolute Shrinkage and Selection Operator
HCC: Hepatocellular carcinoma
RFS: Relapse-free survival
KEGG: Kyoto Encyclopedia of Genes and Genomes
TME: The tumor microenvironment
HBV: Hepatitis B virus
NAT: Normal tissue adjacent to tumor
