# Supplementary material for: A lipid metabolism–based prognostic risk model for HBV–related hepatocellular carcinoma
Source: Lipids Health Dis. 2023 Apr 1;22:46. doi: 10.1186/s12944-023-01780-9 (PMC10067291; doi:10.1186/s12944-023-01780-9)
Supplement: Supplementary file 1 — Additional file 1: Fig. S1. Feature selection by LASSO logistic regression. Fig. S2. The prognostic contributions of eleven marker genes in the risk model. Fig. S3.Survival difference analysis between hbv + and hbv- HCC patients in the TCGA cohort. Fig. S4. Expression patterns comparison of 11 marker genes between hbv + HCC and normal samples. (A-B) The mRNAs expression level of 11 genes in the TCGA and Gao et al. cohorts, respectively. (C) The protein expression level of 11 genes in Gao et al. cohort. Fig. S5. Survival analysis of high- and low-risk groups. Fig. S6. Independent prognostic prediction analysis of our risk model. Fig. S7. Immune cells infiltration difference between high- and low-risk groups quantified by cibersort algorithm. Fig. S8. Functional enrichment analysis. Fig. S9. Analysis of immune gene expression difference in high- and low-risk groups. Fig. S10. TMB and intratumor genetic heterogeneity difference between high- and low-risk groups. [file 12944_2023_1780_MOESM1_ESM.zip › Supplemental Documents.docx]

## Supplemental Documents


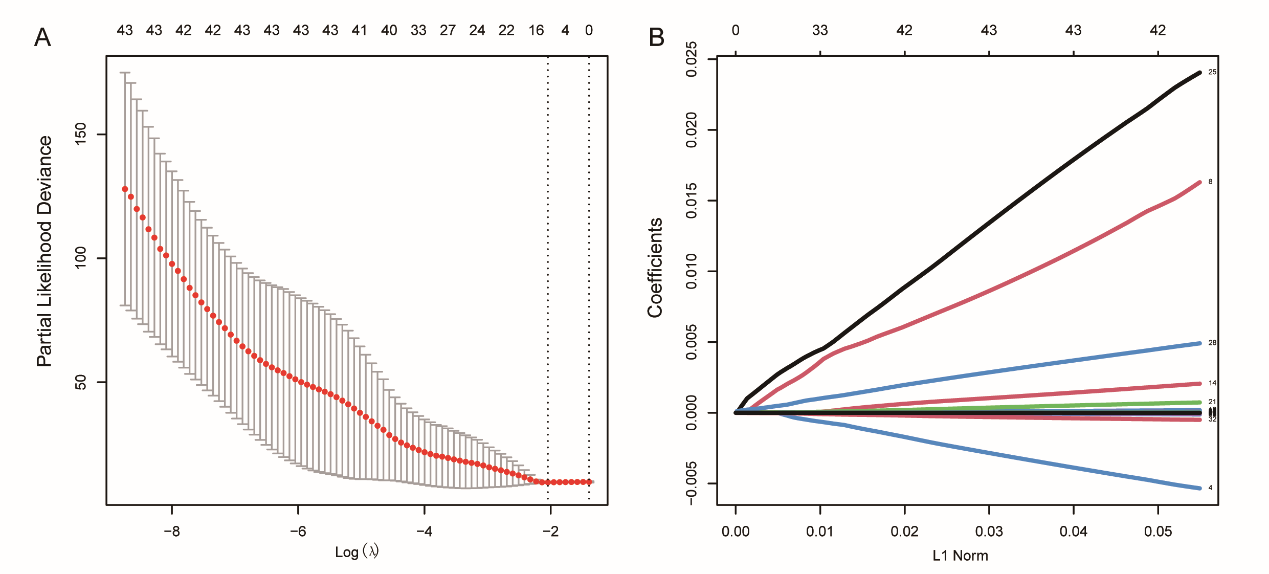
Figure S1. Feature selection by LASSO logistic regression.

(A) The optimal regularization parameters (λ) selection based on 10-fold cross-validation. The partial likelihood deviance value (y-axis) was presented with varied log (λ) (x-axis). The dotted vertical lines delineate minimum criteria and the 1-SE norm criteria. (B) LASSO coefficient sketch of features. LASSO, least absolute shrinkage and selection operator; SE, standard error; λ, lambda.


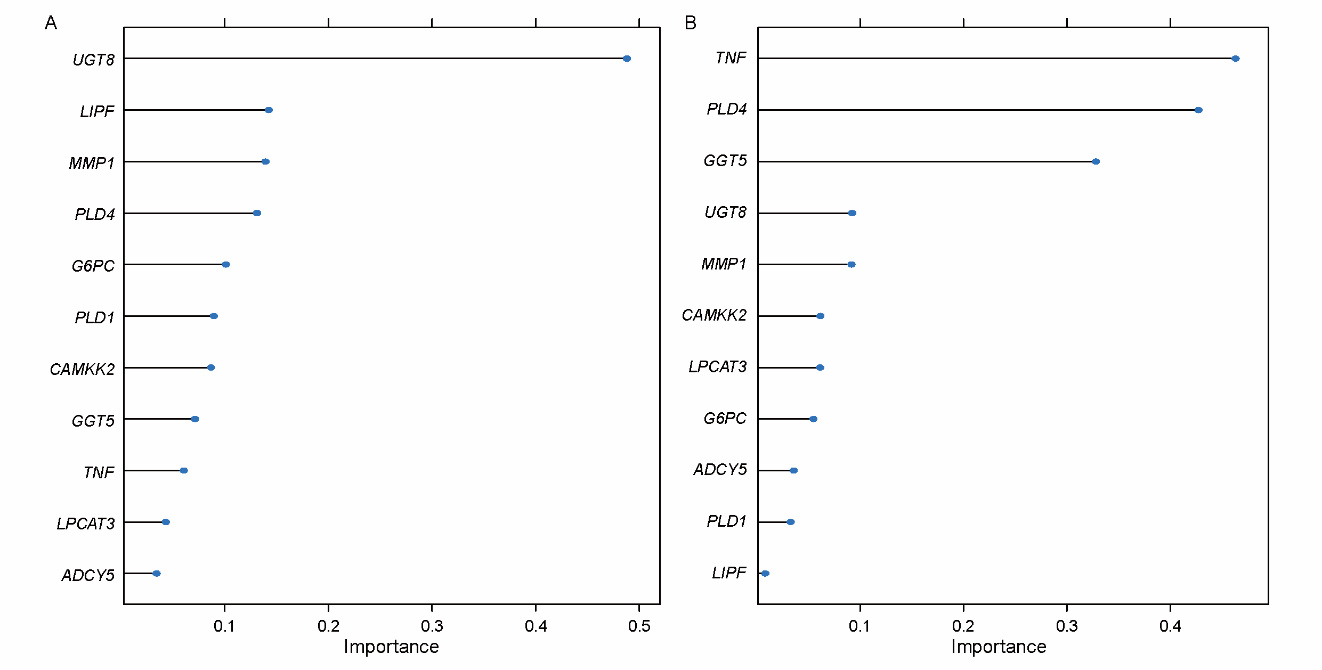


Figure S2. The prognostic contributions of eleven marker genes in the risk model.

The importance of eleven genes for the risk model in the TCGA cohort (A) and the Gao et al. cohort (B).


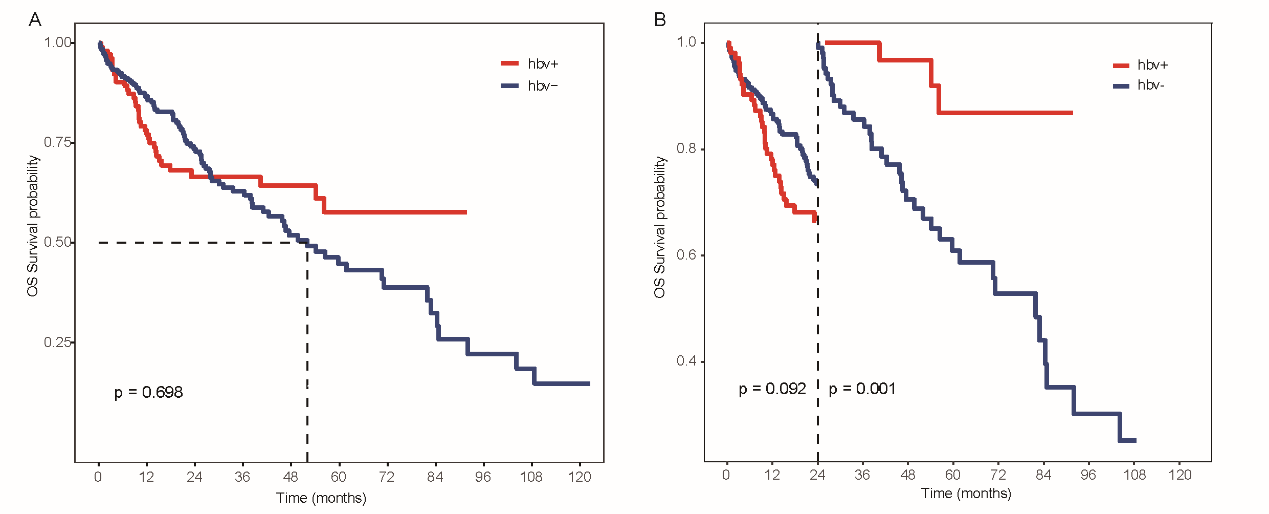


Figure S3. Survival difference analysis between hbv+ and hbv- HCC patients in the TCGA cohort.

(A) OS difference analysis between hbv+ and hbv- HCC patients. (B) Landmark analysis of survival difference between events occurring within and after two years. OS, overall survival; HBV+, hepatitis B virus positive; HBV-, hepatitis B virus negative; HCC, Hepatocellular carcinoma.


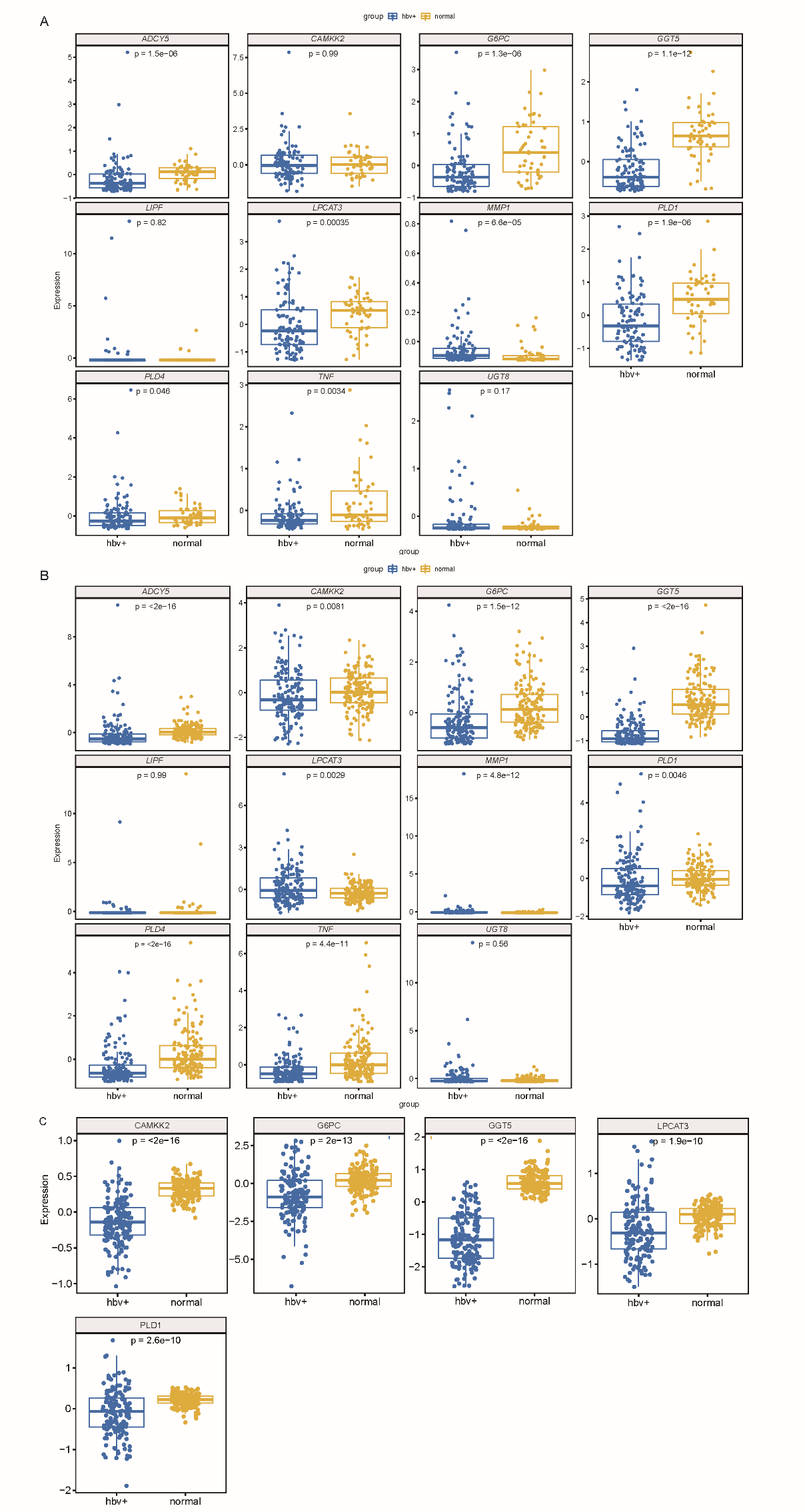


Figure S4. Expression patterns comparison of 11 marker genes between hbv+ HCC and normal samples. (A-B) The mRNAs expression level of 11 genes in the TCGA and Gao et al. cohorts, respectively. (C) The protein expression level of 11 genes in Gao et al. cohort.


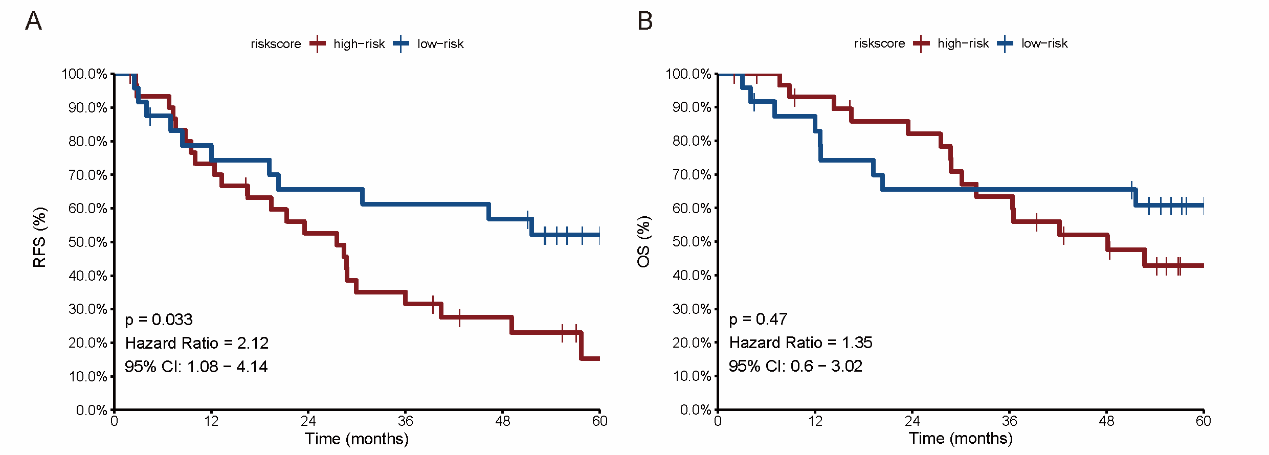


Figure S5. Survival analysis of high- and low-risk groups.

(A) RFS and OS (B) difference between high- and low-risk groups in the Stephanie et al. cohort.

RFS, relapse-free survival; OS, overall survival.


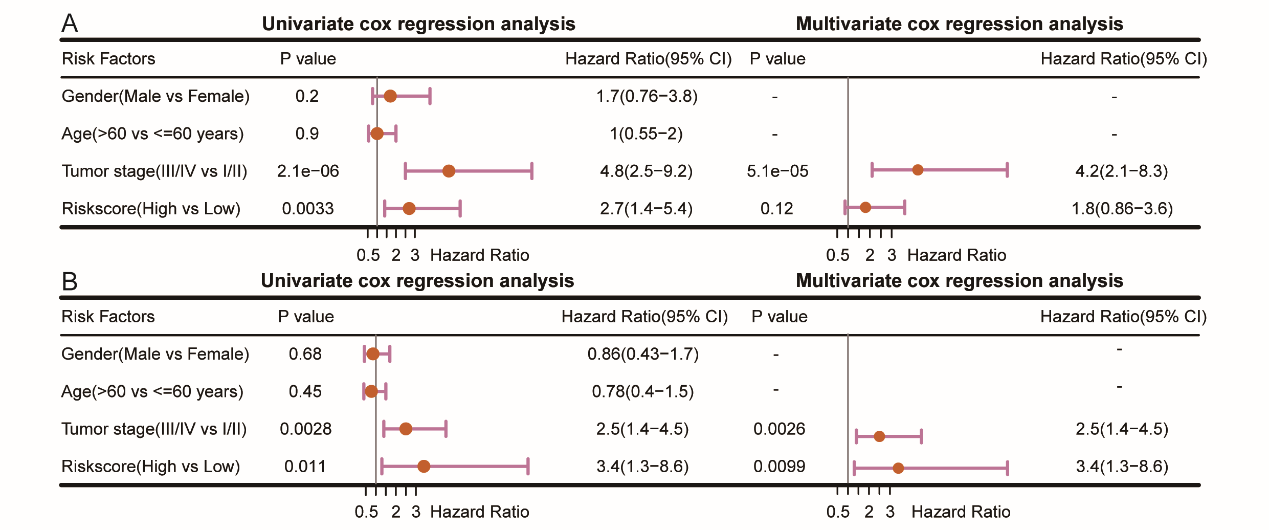


Figure S6. Independent prognostic prediction analysis of our risk model.

(A) Univariate and multivariate Cox regression analysis results based on DFI in HBV HCC patients of the TCGA cohort. (B) Univariate and multivariate Cox regression analysis results based on RFS in HBV HCC patients of Gao et al. cohort. DFI, disease-free interval; RFS, recurrence-free survival; HCC, hepatocellular carcinoma.


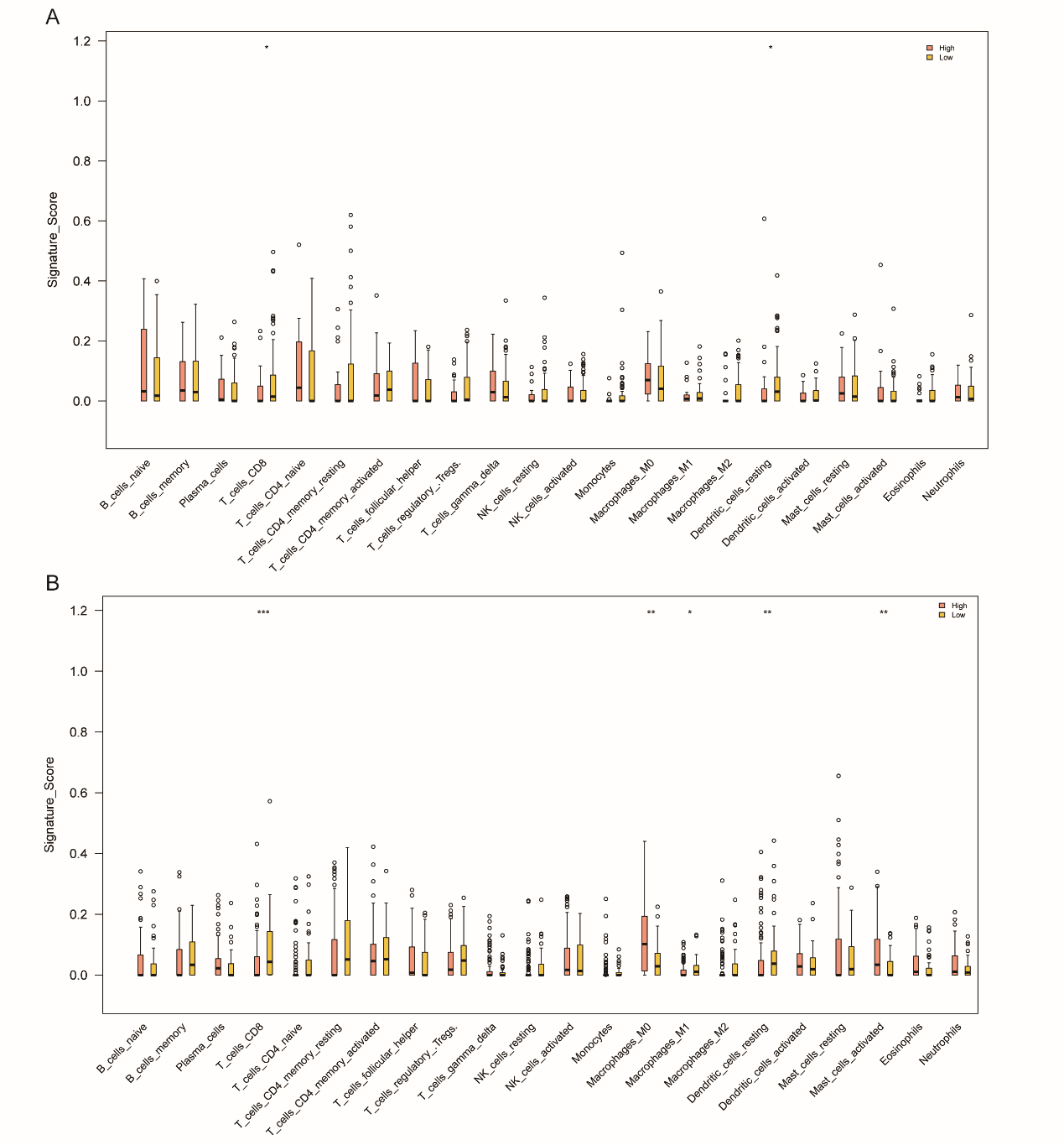


Figure S7. Immune cells infiltration difference between high- and low-risk groups quantified by cibersort algorithm.

(A-B) Significantly different immune cells infiltration box plot of the TCGA and Gao et al. cohorts.

**P* < 0.05; ** *P* < 0.01; *** *P* < 0.001.


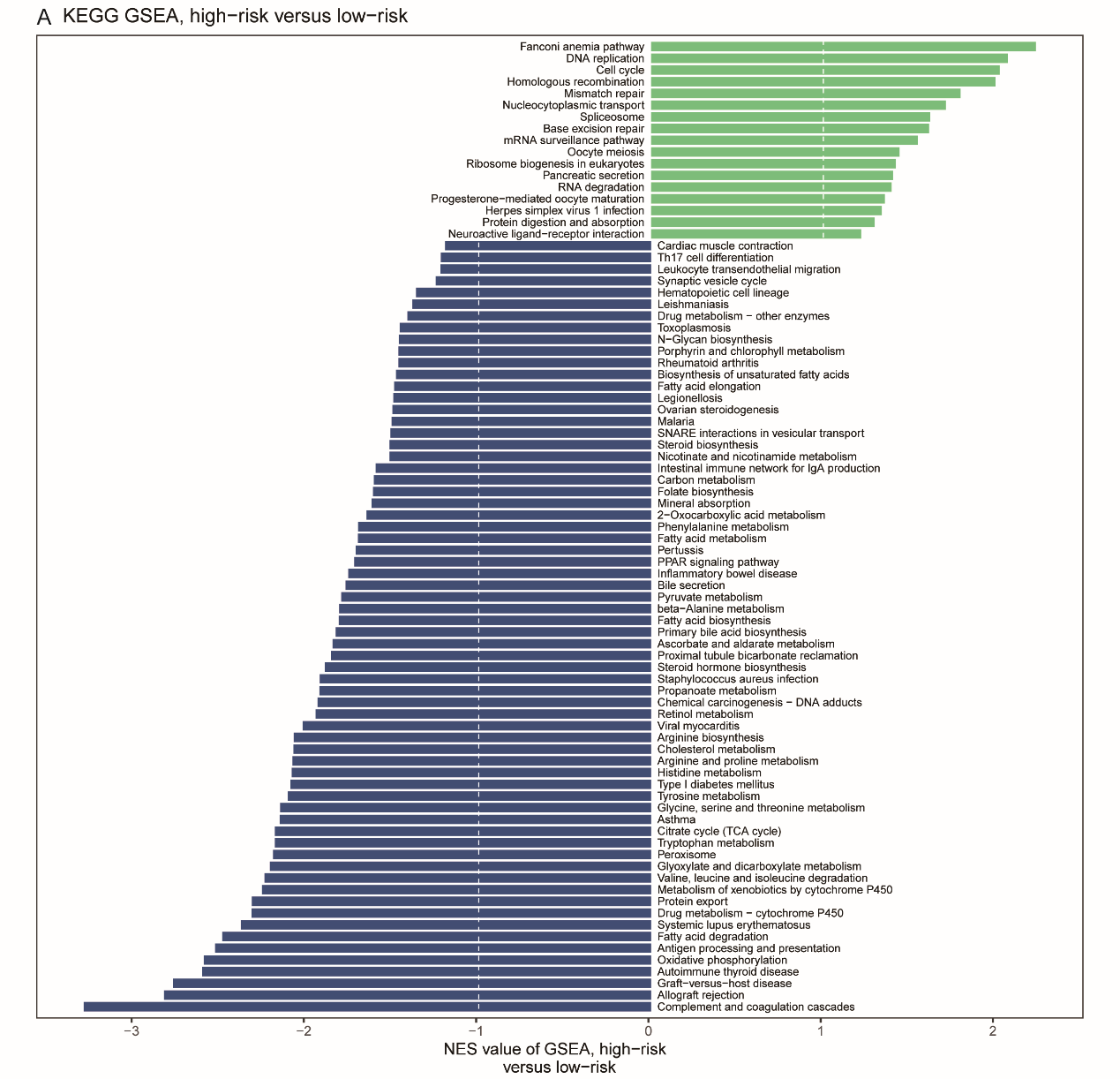


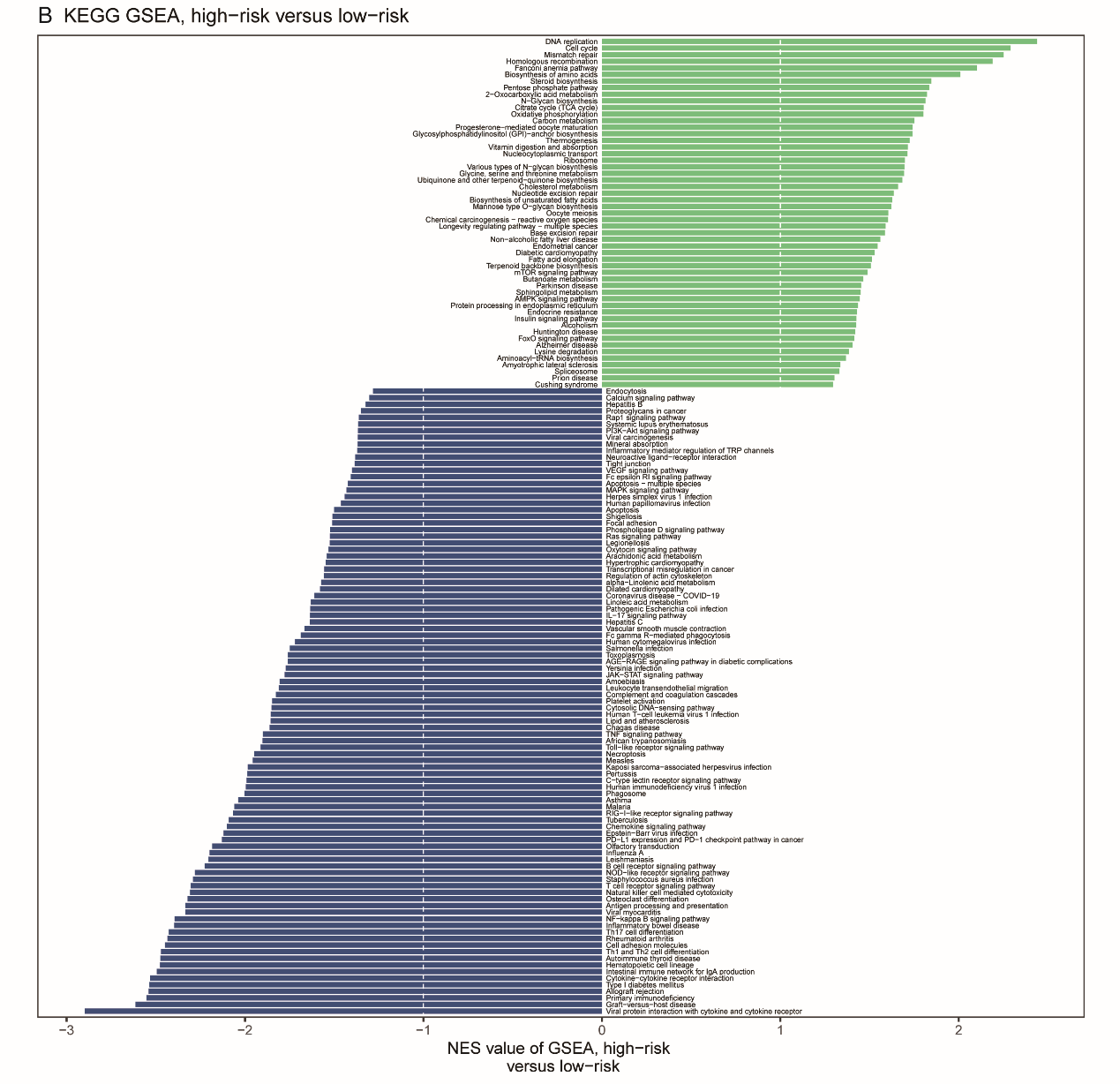


Figure S8. Functional enrichment analysis.

(A-B) Representative and significantly enriched KEGG pathways in high- and low-risk groups of the TCGA and Gao et al. cohorts. (C-D) Hallmark pathway analysis results in high- and low-risk groups of the TCGA and Gao et al. cohorts. Green and blue bars remark significantly enriched pathways in high- and low-risk groups, respectively.


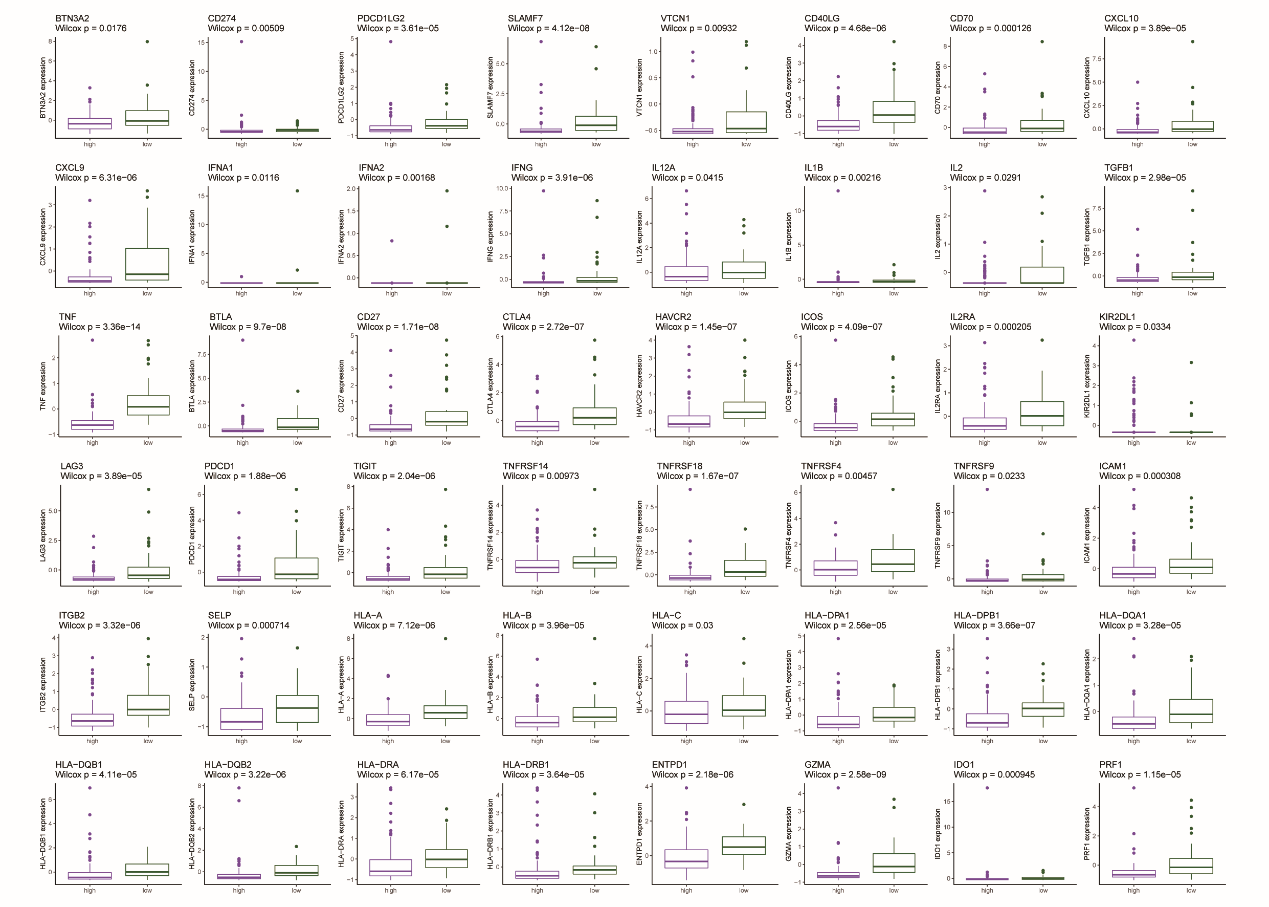


Figure S9. Analysis of immune gene expression difference in high- and low-risk groups.

Box plots of genes with significantly different expression levels between high- and low-risk groups of Gao et al. cohort.


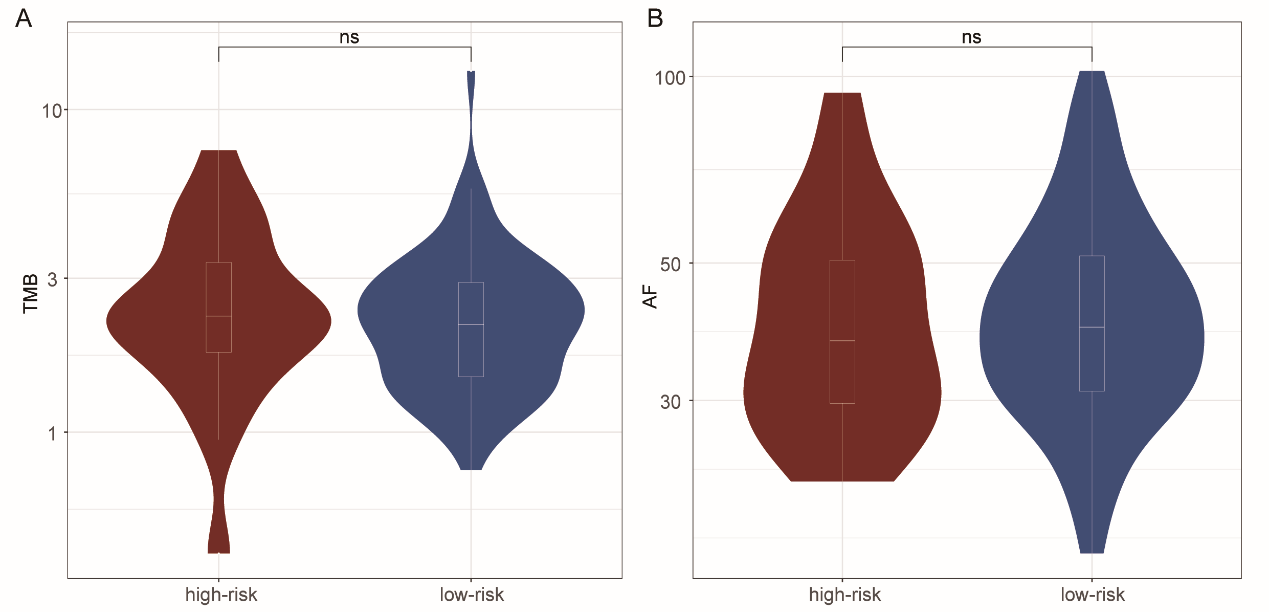


Figure S10. TMB and intratumor genetic heterogeneity difference between high- and low-risk groups.

(A-B) TMB and MATH value in high- and low-risk groups of the TCGA cohort. TMB: tumor mutation burden; MATH: mutant-allele tumor heterogeneity. ns: no significant.
