# Supplementary figures and images for: A lipid metabolism–based prognostic risk model for HBV–related hepatocellular carcinoma
Source: Lipids Health Dis. 2023 Apr 1;22:46. doi: 10.1186/s12944-023-01780-9 (PMC10067291; doi:10.1186/s12944-023-01780-9)

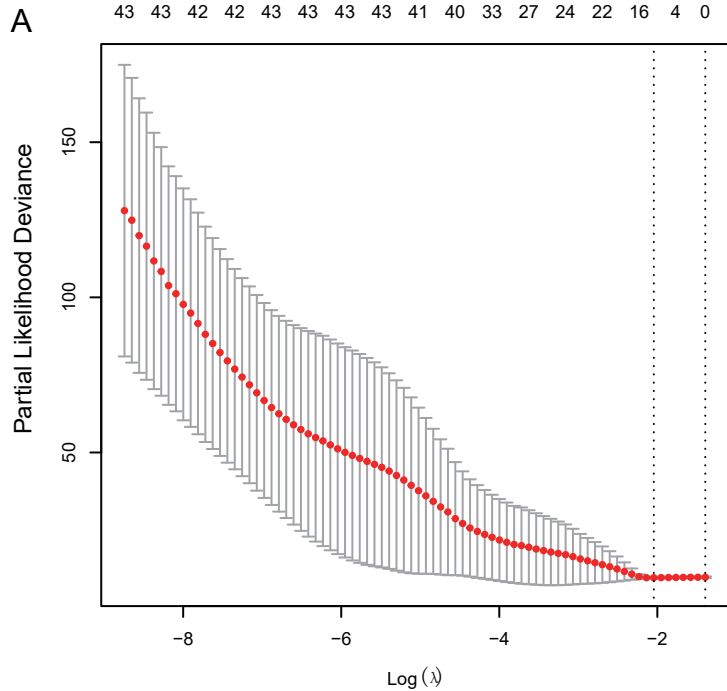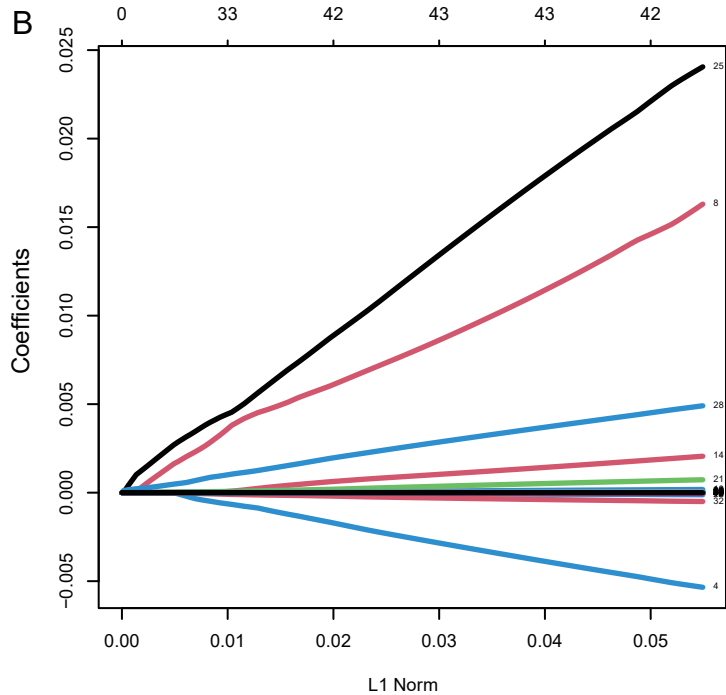

Supplement: Supplementary file 1 — Additional file 1: Fig. S1. Feature selection by LASSO logistic regression. Fig. S2. The prognostic contributions of eleven marker genes in the risk model. Fig. S3.Survival difference analysis between hbv + and hbv- HCC patients in the TCGA cohort. Fig. S4. Expression patterns comparison of 11 marker genes between hbv + HCC and normal samples. (A-B) The mRNAs expression level of 11 genes in the TCGA and Gao et al. cohorts, respectively. (C) The protein expression level of 11 genes in Gao et al. cohort. Fig. S5. Survival analysis of high- and low-risk groups. Fig. S6. Independent prognostic prediction analysis of our risk model. Fig. S7. Immune cells infiltration difference between high- and low-risk groups quantified by cibersort algorithm. Fig. S8. Functional enrichment analysis. Fig. S9. Analysis of immune gene expression difference in high- and low-risk groups. Fig. S10. TMB and intratumor genetic heterogeneity difference between high- and low-risk groups. [file 12944_2023_1780_MOESM1_ESM.zip › Figure S1.pdf]

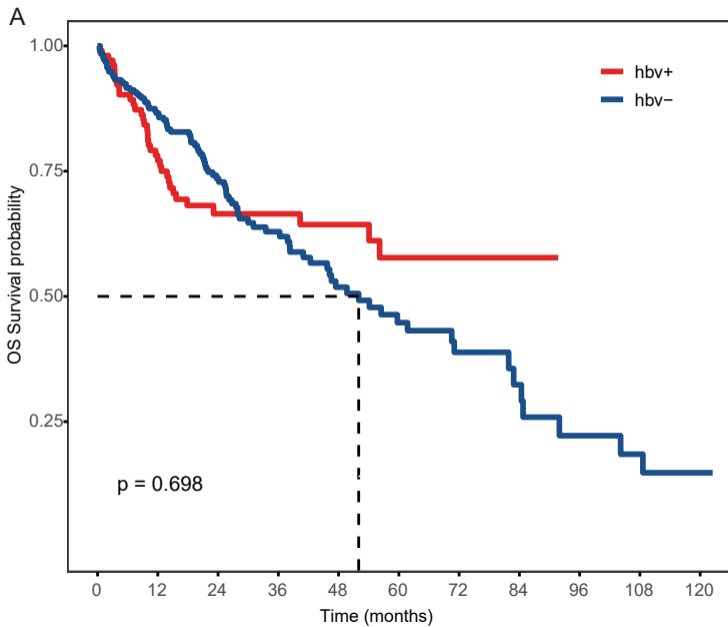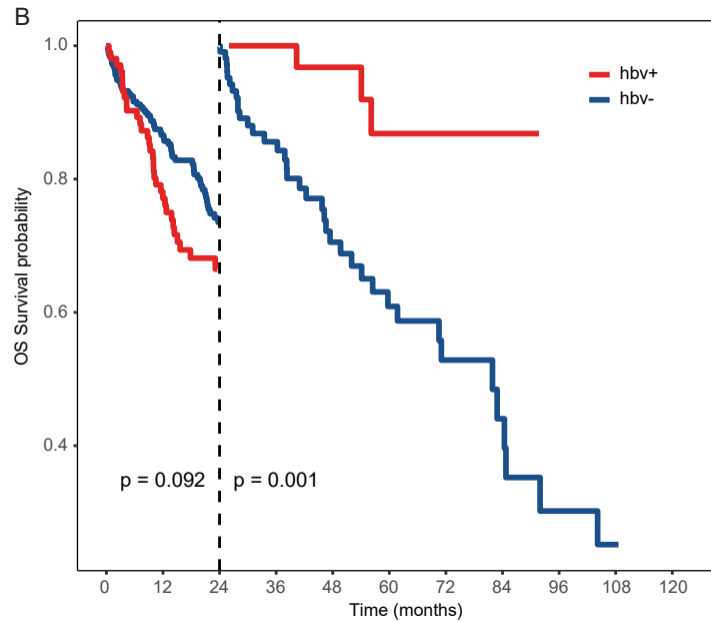

Supplement: Supplementary file 1 — Additional file 1: Fig. S1. Feature selection by LASSO logistic regression. Fig. S2. The prognostic contributions of eleven marker genes in the risk model. Fig. S3.Survival difference analysis between hbv + and hbv- HCC patients in the TCGA cohort. Fig. S4. Expression patterns comparison of 11 marker genes between hbv + HCC and normal samples. (A-B) The mRNAs expression level of 11 genes in the TCGA and Gao et al. cohorts, respectively. (C) The protein expression level of 11 genes in Gao et al. cohort. Fig. S5. Survival analysis of high- and low-risk groups. Fig. S6. Independent prognostic prediction analysis of our risk model. Fig. S7. Immune cells infiltration difference between high- and low-risk groups quantified by cibersort algorithm. Fig. S8. Functional enrichment analysis. Fig. S9. Analysis of immune gene expression difference in high- and low-risk groups. Fig. S10. TMB and intratumor genetic heterogeneity difference between high- and low-risk groups. [file 12944_2023_1780_MOESM1_ESM.zip › Figure S3.pdf]

A

group ■ hbv+ ■ normal

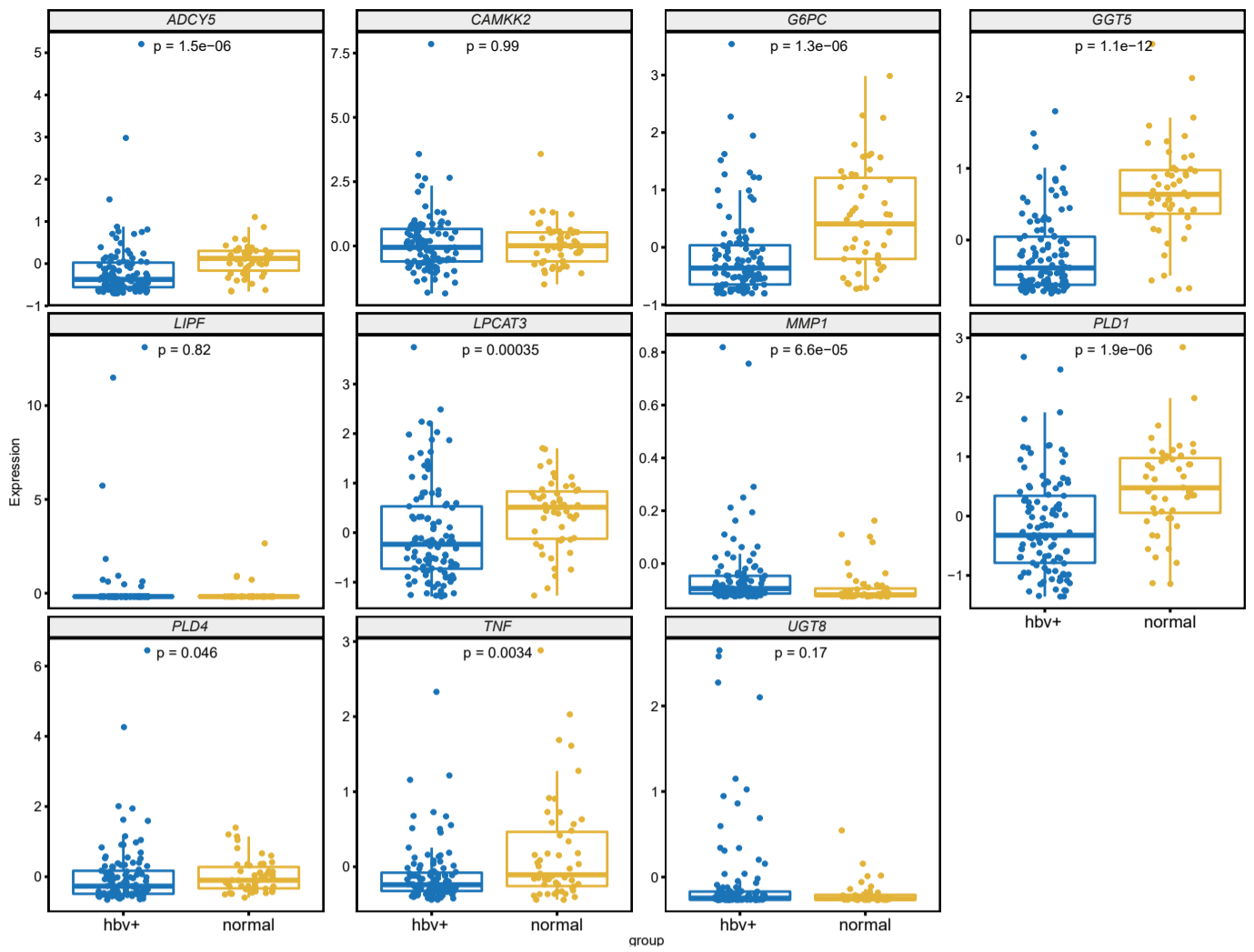

B

group ■ hbv+ ■ normal

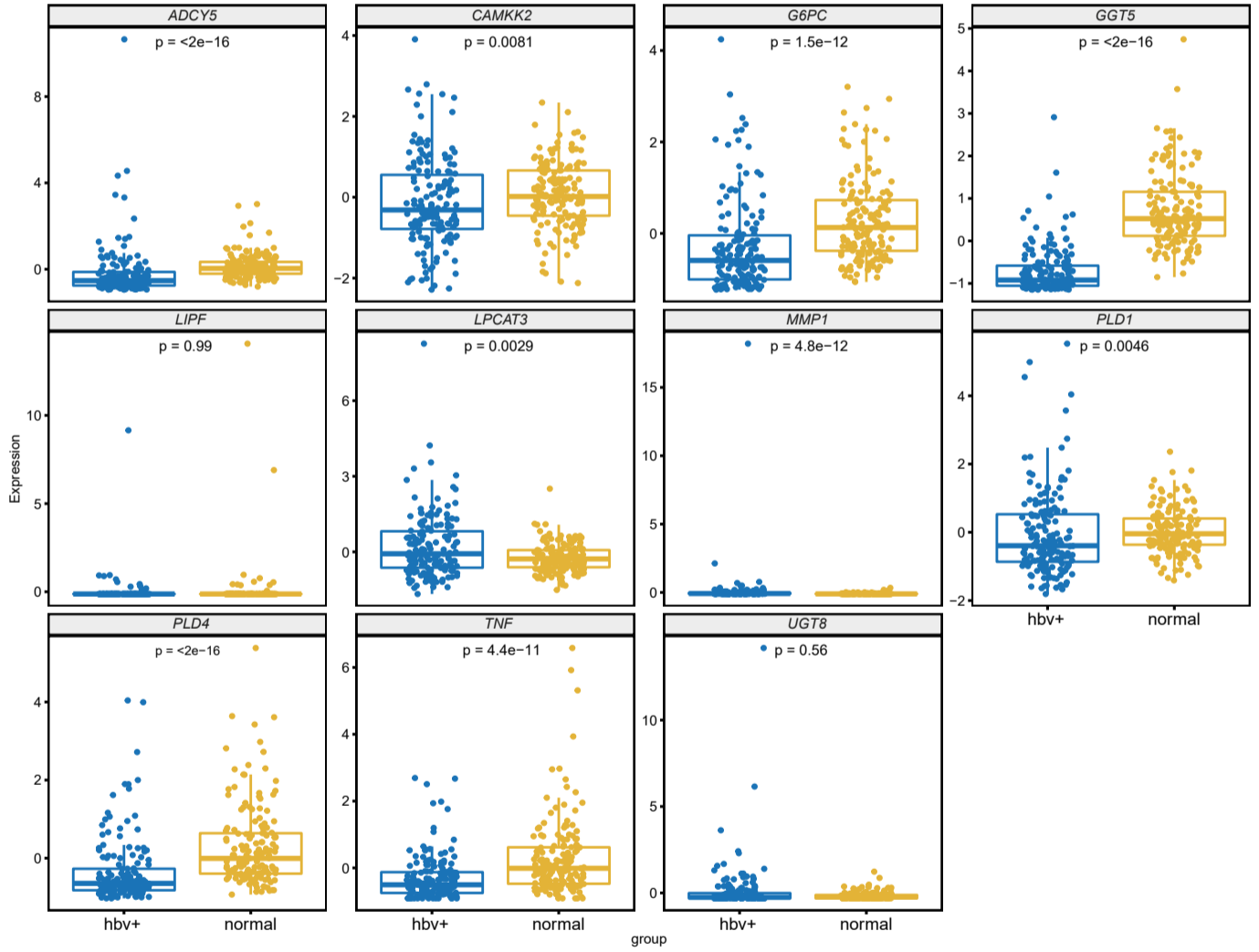

C

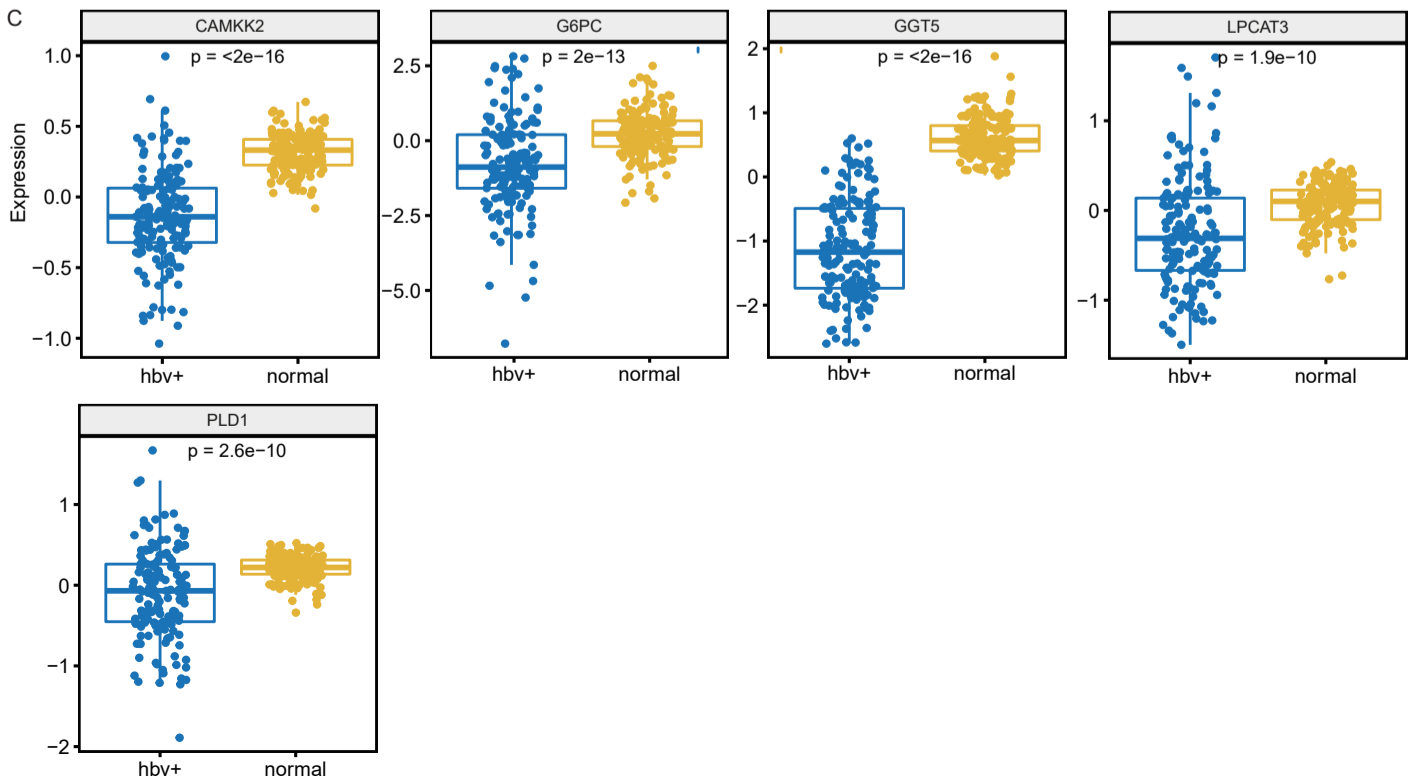

Supplement: Supplementary file 1 — Additional file 1: Fig. S1. Feature selection by LASSO logistic regression. Fig. S2. The prognostic contributions of eleven marker genes in the risk model. Fig. S3.Survival difference analysis between hbv + and hbv- HCC patients in the TCGA cohort. Fig. S4. Expression patterns comparison of 11 marker genes between hbv + HCC and normal samples. (A-B) The mRNAs expression level of 11 genes in the TCGA and Gao et al. cohorts, respectively. (C) The protein expression level of 11 genes in Gao et al. cohort. Fig. S5. Survival analysis of high- and low-risk groups. Fig. S6. Independent prognostic prediction analysis of our risk model. Fig. S7. Immune cells infiltration difference between high- and low-risk groups quantified by cibersort algorithm. Fig. S8. Functional enrichment analysis. Fig. S9. Analysis of immune gene expression difference in high- and low-risk groups. Fig. S10. TMB and intratumor genetic heterogeneity difference between high- and low-risk groups. [file 12944_2023_1780_MOESM1_ESM.zip › Figure S4.pdf]

A

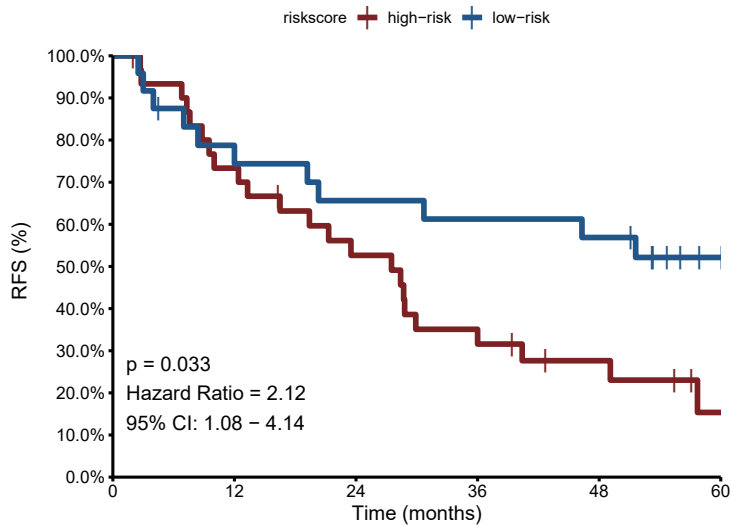

B

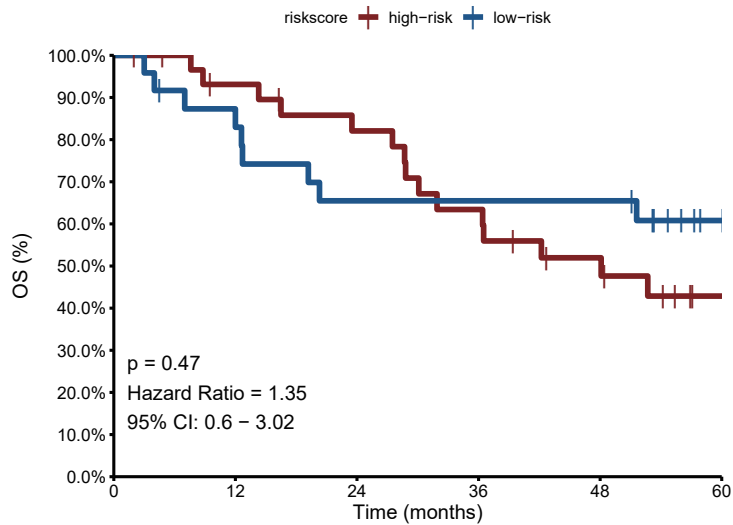

Supplement: Supplementary file 1 — Additional file 1: Fig. S1. Feature selection by LASSO logistic regression. Fig. S2. The prognostic contributions of eleven marker genes in the risk model. Fig. S3.Survival difference analysis between hbv + and hbv- HCC patients in the TCGA cohort. Fig. S4. Expression patterns comparison of 11 marker genes between hbv + HCC and normal samples. (A-B) The mRNAs expression level of 11 genes in the TCGA and Gao et al. cohorts, respectively. (C) The protein expression level of 11 genes in Gao et al. cohort. Fig. S5. Survival analysis of high- and low-risk groups. Fig. S6. Independent prognostic prediction analysis of our risk model. Fig. S7. Immune cells infiltration difference between high- and low-risk groups quantified by cibersort algorithm. Fig. S8. Functional enrichment analysis. Fig. S9. Analysis of immune gene expression difference in high- and low-risk groups. Fig. S10. TMB and intratumor genetic heterogeneity difference between high- and low-risk groups. [file 12944_2023_1780_MOESM1_ESM.zip › Figure S5.pdf]

A

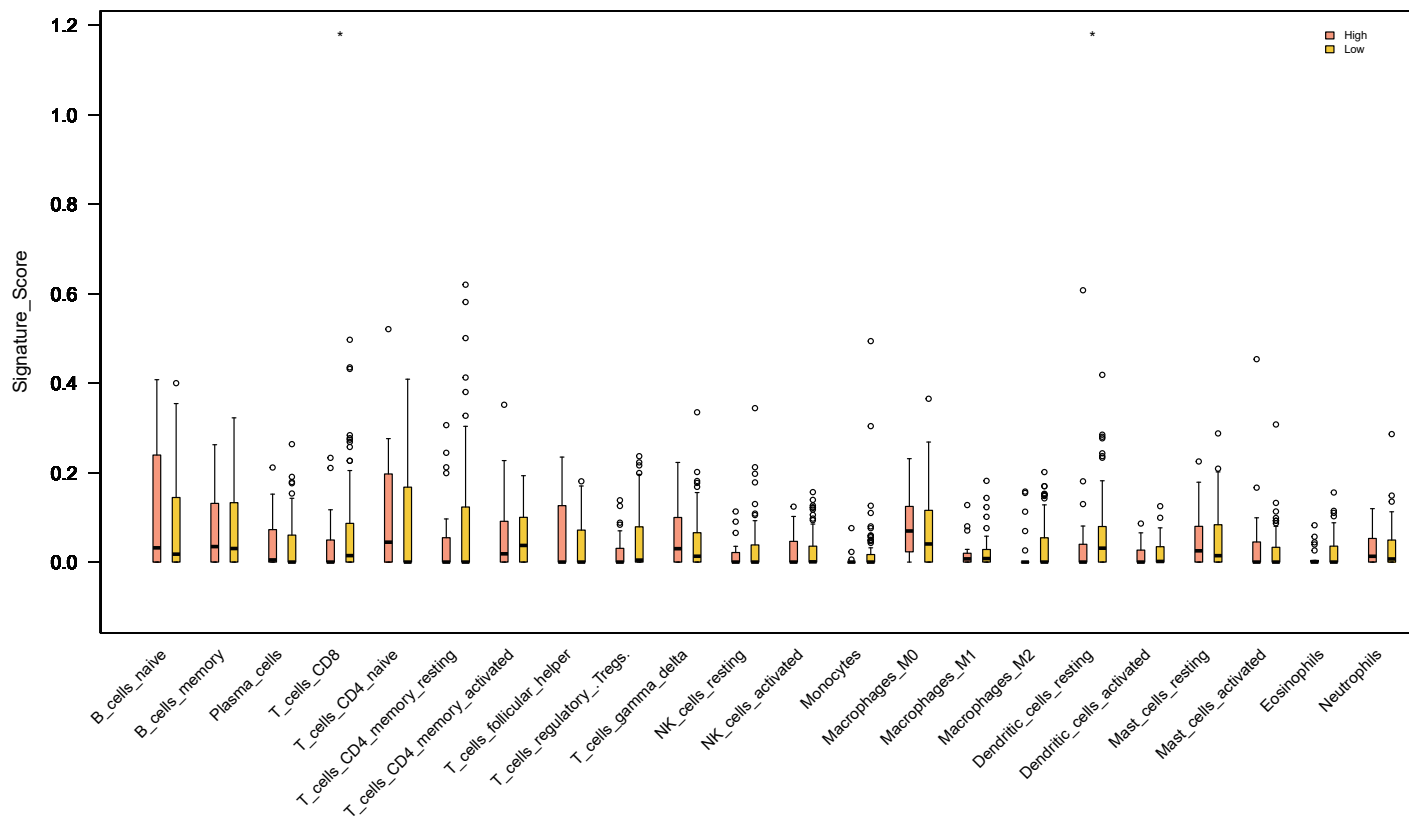

B

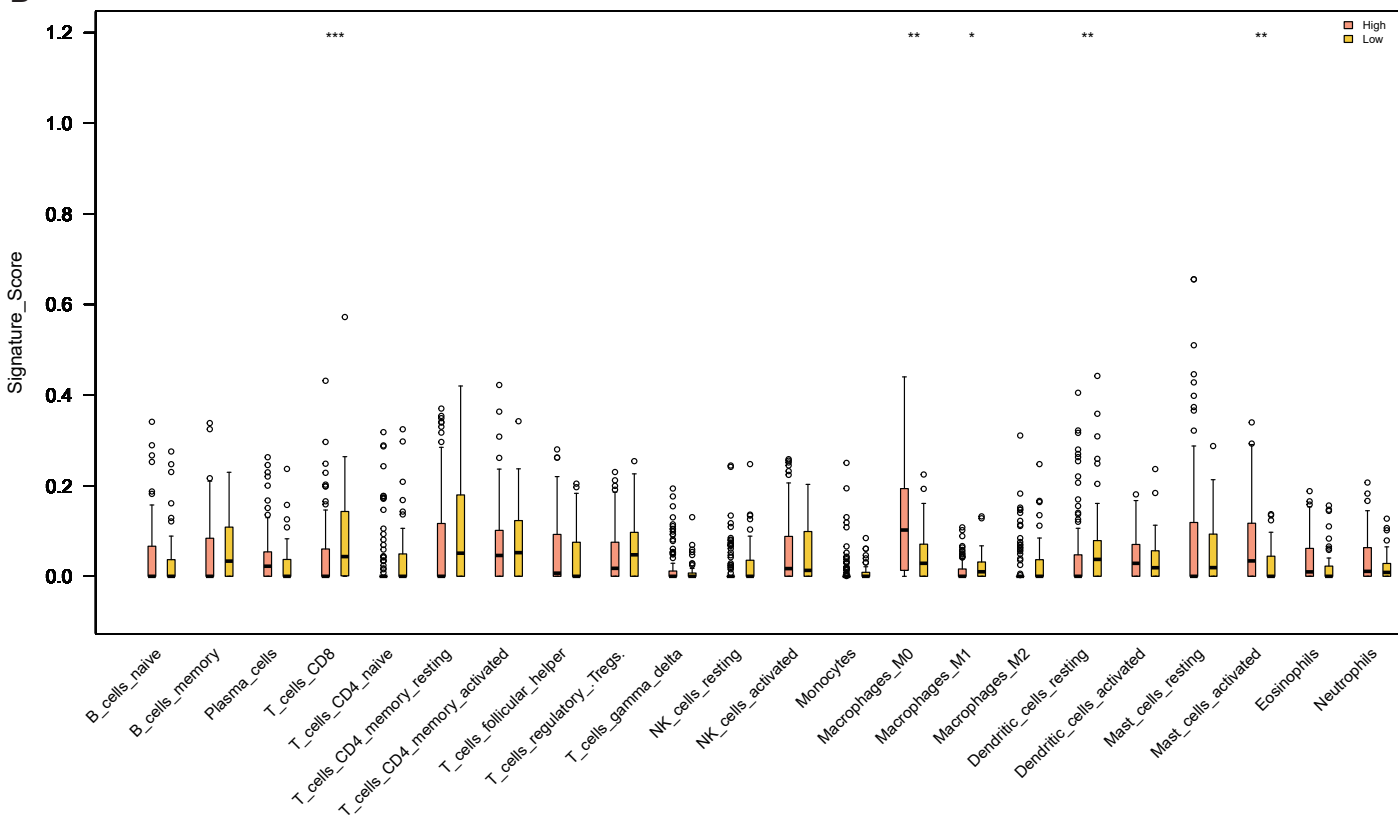

Supplement: Supplementary file 1 — Additional file 1: Fig. S1. Feature selection by LASSO logistic regression. Fig. S2. The prognostic contributions of eleven marker genes in the risk model. Fig. S3.Survival difference analysis between hbv + and hbv- HCC patients in the TCGA cohort. Fig. S4. Expression patterns comparison of 11 marker genes between hbv + HCC and normal samples. (A-B) The mRNAs expression level of 11 genes in the TCGA and Gao et al. cohorts, respectively. (C) The protein expression level of 11 genes in Gao et al. cohort. Fig. S5. Survival analysis of high- and low-risk groups. Fig. S6. Independent prognostic prediction analysis of our risk model. Fig. S7. Immune cells infiltration difference between high- and low-risk groups quantified by cibersort algorithm. Fig. S8. Functional enrichment analysis. Fig. S9. Analysis of immune gene expression difference in high- and low-risk groups. Fig. S10. TMB and intratumor genetic heterogeneity difference between high- and low-risk groups. [file 12944_2023_1780_MOESM1_ESM.zip › Figure S7.pdf]

A KEGG GSEA, high-risk versus low-risk

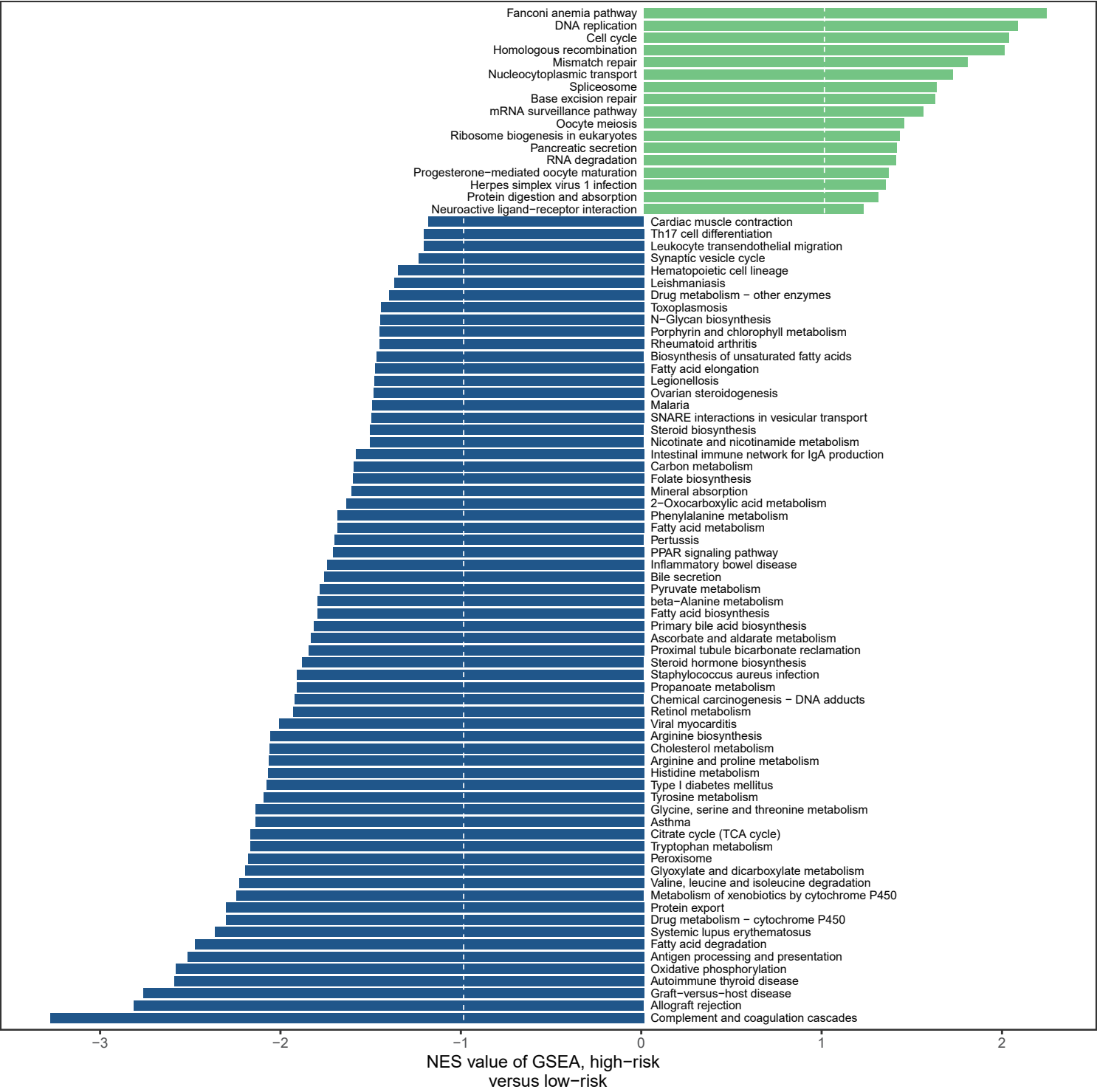

B KEGG GSEA, high-risk versus low-risk

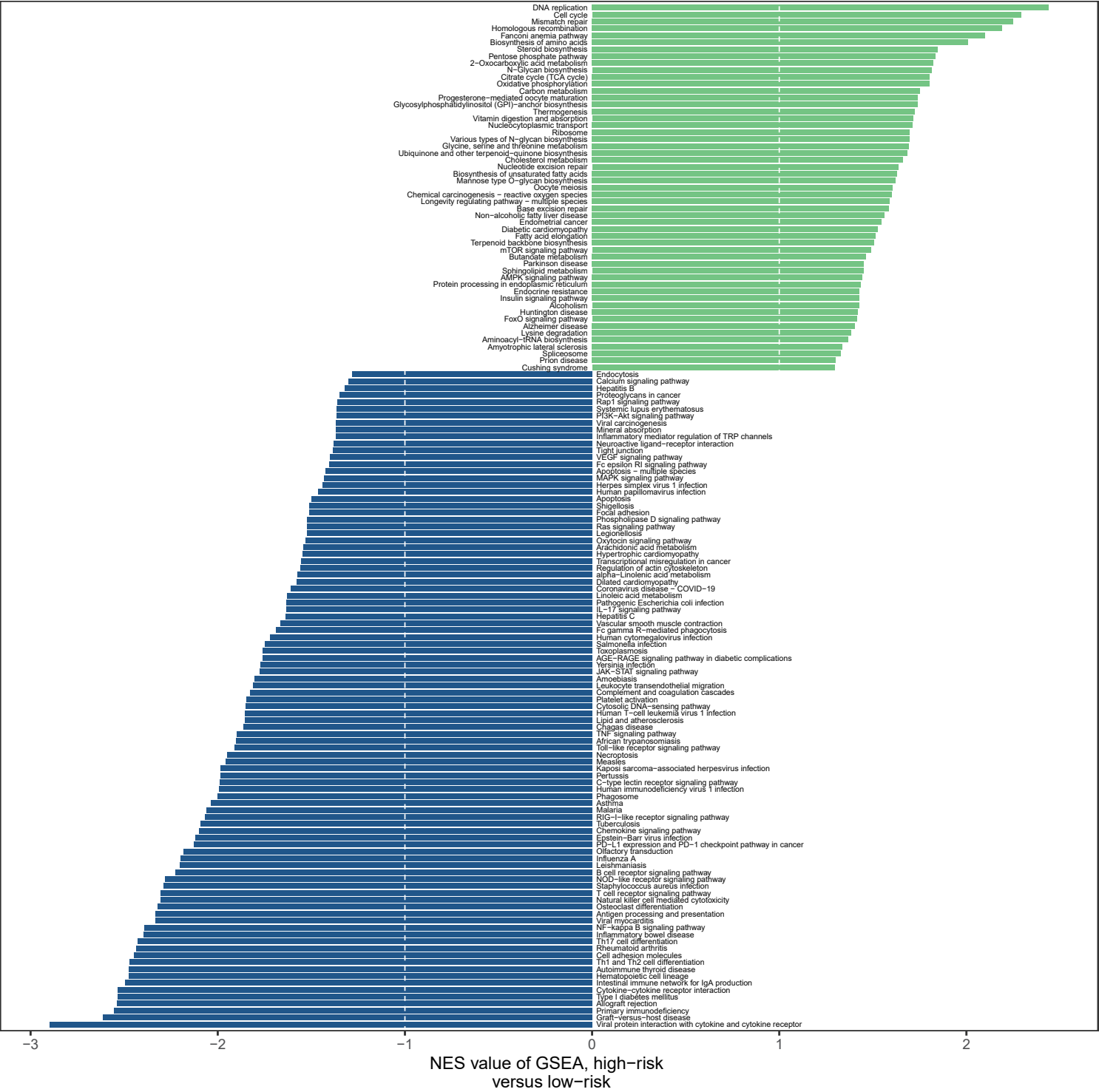

Supplement: Supplementary file 1 — Additional file 1: Fig. S1. Feature selection by LASSO logistic regression. Fig. S2. The prognostic contributions of eleven marker genes in the risk model. Fig. S3.Survival difference analysis between hbv + and hbv- HCC patients in the TCGA cohort. Fig. S4. Expression patterns comparison of 11 marker genes between hbv + HCC and normal samples. (A-B) The mRNAs expression level of 11 genes in the TCGA and Gao et al. cohorts, respectively. (C) The protein expression level of 11 genes in Gao et al. cohort. Fig. S5. Survival analysis of high- and low-risk groups. Fig. S6. Independent prognostic prediction analysis of our risk model. Fig. S7. Immune cells infiltration difference between high- and low-risk groups quantified by cibersort algorithm. Fig. S8. Functional enrichment analysis. Fig. S9. Analysis of immune gene expression difference in high- and low-risk groups. Fig. S10. TMB and intratumor genetic heterogeneity difference between high- and low-risk groups. [file 12944_2023_1780_MOESM1_ESM.zip › Figure S8.pdf]

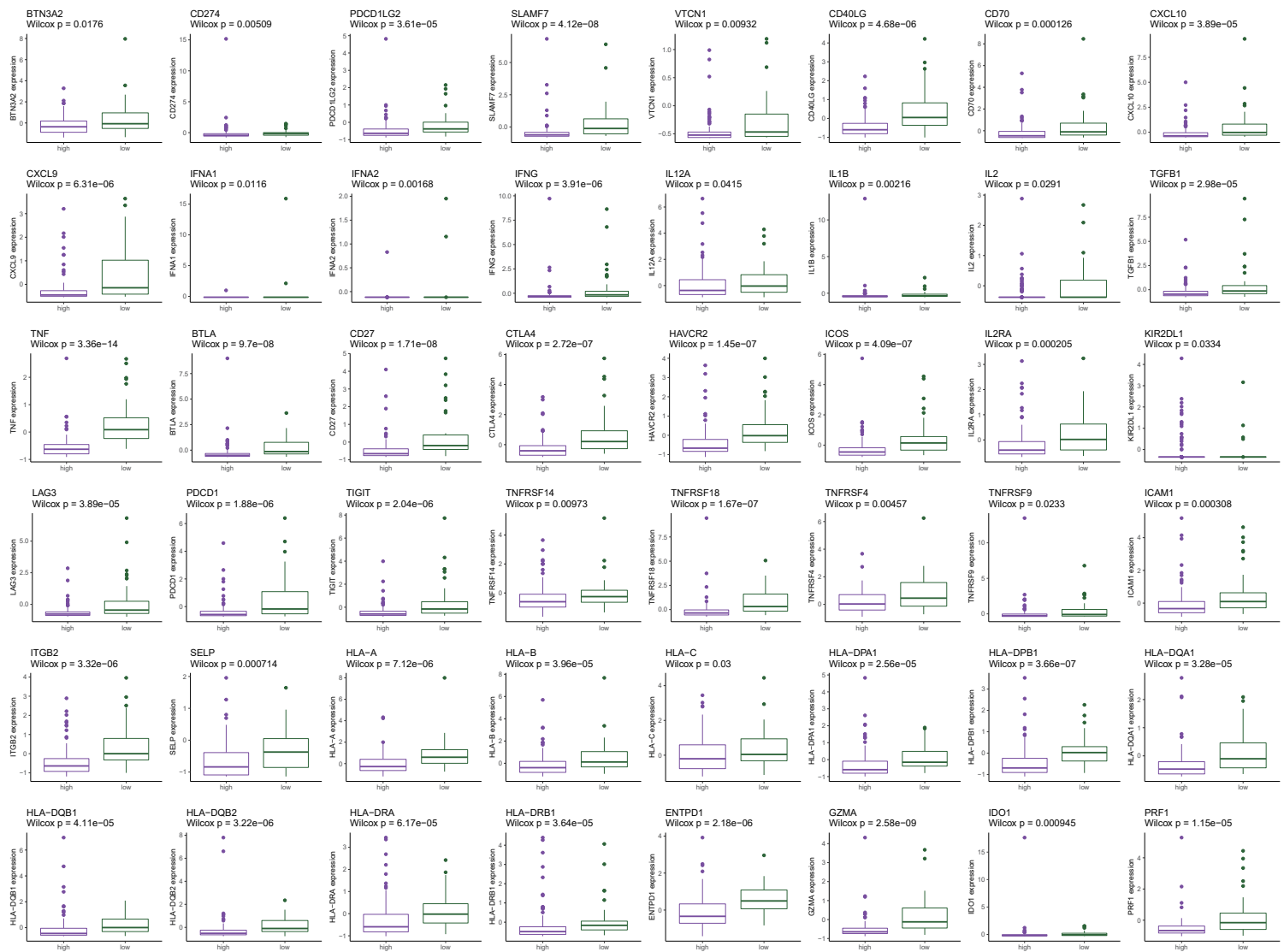

Supplement: Supplementary file 1 — Additional file 1: Fig. S1. Feature selection by LASSO logistic regression. Fig. S2. The prognostic contributions of eleven marker genes in the risk model. Fig. S3.Survival difference analysis between hbv + and hbv- HCC patients in the TCGA cohort. Fig. S4. Expression patterns comparison of 11 marker genes between hbv + HCC and normal samples. (A-B) The mRNAs expression level of 11 genes in the TCGA and Gao et al. cohorts, respectively. (C) The protein expression level of 11 genes in Gao et al. cohort. Fig. S5. Survival analysis of high- and low-risk groups. Fig. S6. Independent prognostic prediction analysis of our risk model. Fig. S7. Immune cells infiltration difference between high- and low-risk groups quantified by cibersort algorithm. Fig. S8. Functional enrichment analysis. Fig. S9. Analysis of immune gene expression difference in high- and low-risk groups. Fig. S10. TMB and intratumor genetic heterogeneity difference between high- and low-risk groups. [file 12944_2023_1780_MOESM1_ESM.zip › Figure S9.pdf]
